# *Tim1* Deficiency Mediates Gestational Hyperglycemia‐Related Syncytiotrophoblast Dysfunction and Fetal Growth Restriction

**DOI:** 10.1002/advs.202508686

**Published:** 2026-02-11

**Authors:** Junsen She, Rui Liu, Chen Fang, Shuyu Zhang, Yizhi Hu, Fei Guo, Yuhang Long, Mengzhen Ding, Haiyan Wu, Bokang Zhou, Zexin Yang, Ying Jiang, Jianzhong Sheng, Ling Gao, Hefeng Huang

**Affiliations:** ^1^ Center for Reproductive Medicine International Institutes of Medicine The Fourth Affiliated Hospital Zhejiang University School of Medicine Yiwu China; ^2^ Women's Hospital Key Laboratory of Reproductive Genetics (Ministry of Education) Zhejiang University School of Medicine Hangzhou China; ^3^ The International Peace Maternity and Child Health Hospital School of Medicine Shanghai Jiao Tong University Shanghai China; ^4^ Obstetrics and Gynecology Hospital Institute of Reproduction and Development Fudan University Shanghai China; ^5^ Research Units of Embryo Original Diseases Chinese Academy of Medical Sciences Shanghai China

**Keywords:** fetal growth restriction, gestational hyperglycemia, oxidative stress, syncytiotrophoblast

## Abstract

Gestational hyperglycemia (GHG) causes fetal growth restriction (FGR), while its mechanism remains incompletely understood. Defects in the syncytiotrophoblast, which is pivotal for maternal‐fetal substance exchange, adversely affect fetal development. Whether dysfunction in syncytiotrophoblast is involved in GHG‐related FGR remains unclear. In this study, we used an STZ‐induced GHG mouse model and found that GHG‐induced FGR (44.5% reduction in fetal weight) was associated with a 28.3% decrease in placental efficiency. Immunofluorescence and transmission electron microscopy examinations revealed defective formation of the syncytiotrophoblast layer in GHG placenta, resulting from impaired fusion of trophoblast cells. Gene expression profiling and staining analysis of the placenta revealed that *Tim1*, a phosphatidylserine‐binding protein, was 43.5% downregulated in GHG placenta. In vitro studies confirmed that hyperglycemia decreased *Tim1* and led to trophoblast fusion defects. *Tim1* silence alone recapitulated the effects of hyperglycemia on trophoblast fusion, while *Tim1* overexpression rescued the anti‐fusion effects of hyperglycemia. Moreover, we generated a *Tim1* knockout mouse strain, and observed that *Tim1* deficiency alone induced defective formation of syncytiotrophoblast and FGR during pregnancy. Further analysis revealed that *Tim1* was downregulated by hyperglycemia‐related oxidative stress. Antioxidant treatment during pregnancy reversed *Tim1* downregulation, promoted syncytiotrophoblast formation and improved FGR. Finally, the reduction of *TIM1* expression was confirmed in human placenta with pre‐gestational diabetes and FGR. These findings suggest that *Tim1* downregulation in GHG inhibits placental syncytiotrophoblast formation and contributes to FGR.

## Graphical abstract

Hyperglycemia during pregnancy impairs the fusion of trophoblast cells into syncytiotrophoblasts, leading to fetal growth restriction. This impaired fusion is mediated by *Tim1* downregulation via hyperglycemia‐induced ROS. Antioxidant therapy during pregnancy promotes syncytiotrophoblast formation by upregulating *Tim1* expression, thus alleviating fetal growth restriction.

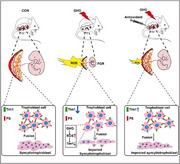

## 1 Introduction

Gestational hyperglycemia (GHG) is prevalent, consisting of gestational diabetes mellitus (GDM) and pregestational diabetes mellitus (PGDM) [1]. GDM is characterized by its first diagnosis during the second or third trimester without a prior history of diabetes, whereas PGDM denotes pre‐existing diabetes that results in persistent hyperglycemia throughout pregnancy [2, 3]. Both GDM and PGDM are associated with adverse maternal and fetal outcomes; however, PGDM exerts a more profound impact on early placental development and fetal organogenesis, contributing to placental structural abnormalities [4] and fetal growth restriction (FGR) [5, 6]. FGR increases the risk of intrauterine demise and neonatal mortality [7], and predisposes offspring to an elevated risk of chronic diseases later in life [8], similar to other early‐life adverse·exposures [9]. However, the mechanisms of FGR caused by GHG remain unclear.

The placenta is crucial for the growth and development of offspring. The placenta performs the function of gas and substance exchange between the mother and the fetus [10]. The placenta of mice is divided into three regions: labyrinth zone, junctional zone, and maternal decidua. The placental labyrinth zone mainly contains sinus trophoblast giant cells, two kinds of multinucleated syncytiotrophoblast layers (SynT‐I and SynT‐II), and endothelial cells [11]. The syncytiotrophoblasts are the key structures in the placenta for maternal‐fetal substance exchange [12]. Previous studies have shown that inhibition of syncytiotrophoblast formation could induce fetal death [13]. Defects in the syncytiotrophoblast layers are associated with developmental heart diseases in mice [14]. Thus, syncytiotrophoblast plays a crucial role in fetal growth and development. However, it is still unclear whether syncytiotrophoblast is impaired in GHG and further contributes to FGR.

In this study, we aimed to determine whether persistent hyperglycemia from early pregnancy affects the development of the fetus, by influencing the formation of the syncytiotrophoblast and to explore its underlying mechanism, using a STZ‐induced mouse model mimicking PGDM.

## 2 Methods

### 2.1 Animal Studies

**FIGURE 1:**
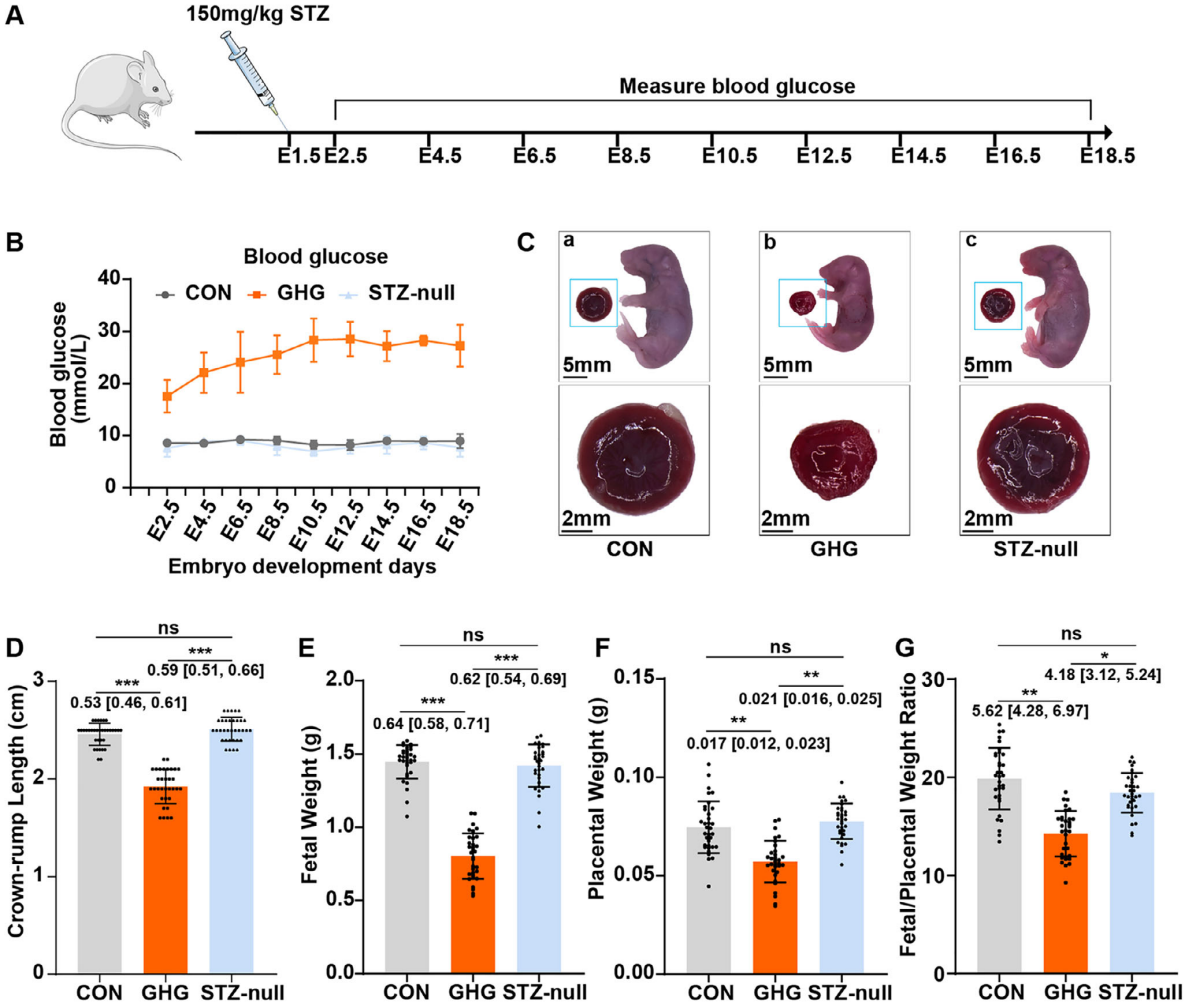
Gestational hyperglycemia leads to fetal growth restriction and placental impairment. (A) Experimental protocol: The day on which the copulation plug was observed in mice was designated as E0.5. After a 12 h fasting period, mouse dams received an intraperitoneal injection of streptozotocin (STZ) at a dose of 150 mg/kg or sodium citrate buffer on E1.5. Starting from E2.5, blood glucose levels were measured every other day. (B) After STZ injection, mice with blood glucose concentrations exceeding 18 mm were assigned to the GHG group. Mice injected with sodium citrate buffer and maintaining normal blood glucose concentrations were assigned to the CON group. Mice with normal blood glucose levels following STZ injection were assigned to the STZ‐null group (*n* = 3–4 dams per group). (C) Comparison of the gross characteristics of the fetuses and placentas among the three groups at E18.5. (D) The crown‐rump length of the E18.5 fetuses from the CON, GHG, and STZ‐null groups (*n* = 33–34 fetuses from 3–4 dams per group). (E) The fetal weight of the E18.5 fetuses from the CON, GHG, and STZ‐null groups (*n* = 33–34 fetuses from 3–4 dams per group). (F) The placental weight of the E18.5 placentas from the CON, GHG, and STZ‐null groups (*n* = 33–34 placentas from 3–4 dams per group). (G) The placental efficiency of the CON, GHG, and STZ‐null groups at E18.5 (*n* = 33–34 placentas from 3 to 4 dams per group). The data are shown as mean ± SD. Multiple fetuses and placentas from the same dam were considered non‐independent and analyzed using a linear mixed model with dam as a random intercept (D–G). The mean difference between the two groups, along with the 95% confidence interval, is displayed beneath the significance bar (D–G). “*n*” indicates the number of independent biological replicates; technical replicates are averaged within each biological replicate before statistical analysis and are not included in the “*n*” values. *p* < 0.05 was considered statistically significant. ^*^ *p* < 0.05, ^**^ *p* < 0.01, ^***^ *p* < 0.001.

The Institute of Cancer Research (ICR) mice were used in the current study. The method for establishing the GHG model was modified from the previous experiment of our team [15]. The day with the presence of a vaginal plug was defined as day 0.5 of embryonic development after fertilization (E0.5). After fasting for 12 h, pregnant mice received intraperitoneal injections of streptozotocin (STZ; 150 mg/kg) and sodium citrate buffer at E1.5, respectively. Pregnant mice were measured for blood glucose levels at multiple gestational timepoints (Figure 1A).

### 2.2 Human Studies

Placenta tissues were procured from deliveries at the Fourth Affiliated Hospital, Zhejiang University School of Medicine. The study has been approved by the Ethics Committee of the Fourth Affiliated Hospital, Zhejiang University School of Medicine, and informed consent has been garnered from all participants. Detailed information is provided in the Online Supporting Information.

### 2.3 Statistical Analysis

Any data from pregnant dams that developed severe distress (e.g., hunched posture), miscarriage, or profound undernutrition following STZ administration were excluded from all subsequent analyses. Power analysis was conducted using PASS software to determine the required number of biological replicates. Continuous data are presented as mean ± standard deviations (SD), and categorical variables are shown as the number (percentage). For all datasets, statistical test assumptions were assessed prior to hypothesis testing. Normality was evaluated using the Shapiro–Wilk test, and variance homogeneity was assessed using Levene's test. To compare the means between two groups, if the data met both normality and variance homogeneity assumptions, an unpaired t‐test is used; otherwise, the Mann–Whitney U test is used. To compare means among multiple groups (more than two) with only one independent variable, a one‐way analysis of variance (ANOVA) is used followed by the Tukey's post hoc analysis if the variances are homogeneous; otherwise, a Welch ANOVA test is employed followed by Games–Howell post hoc analysis. For comparisons of continuous data involving two independent variables, two‐way ANOVA was performed followed by Tukey's post hoc analysis, and data were transformed by square‐root when assumptions were violated. Linear mixed models were used with treatment group as a fixed effect and dam/litter as a random intercept to account for within‐litter non‐independence. Bivariate comparisons of categorical variables were performed using the Chi‐squared test. Correlation analyses were performed using Pearson correlation coefficients. For all statistical analyses, the “*n*” values indicate the number of independent biological replicates. Technical replicates were averaged within each biological replicate before statistical analysis and are not included in the *“n*” values. All statistical analyses were performed using R version 4.4.1 (R Foundation for Statistical Computing). Sample sizes (n) for each analysis are specified in the corresponding figure legends. A *p*‐value (two‐sided) < 0.05 was deemed significant.

### 2.4 Data and Resource Availability

Data are available upon request.

The Expanded Research Design and Methods in the Online Supporting Information include detailed descriptions of the following: human studies, antioxidant therapy in mice, generation of T cell immunoglobulin and mucin domain 1 (*Tim1*) knockout mice, cell culture, determination of cellular oxidative stress, placenta histology analysis, knockdown using specific siRNA, overexpression of TIM1, quantitative Real‐time PCR, western blot, transmission electron microscope, RNA sequencing, and cell fusion assay.

## 3 Results

### 3.1 Gestational Hyperglycemia‐Induced Fetal Growth Restriction is Associated With Placental Impairment

To assess the growth status of fetuses and placentas exposed to hyperglycemia, we used a mouse model of GHG by intraperitoneal injection of STZ (Figure 1A). The STZ model exhibited persistent hyperglycemia from E2.5 (blood glucose > 18 mmol/L; Figure 1B), closely mimicking human pregestational diabetes mellitus (PGDM), which is defined by random plasma glucose ≥ 11.1 mmol/L, rather than the milder hyperglycemia characteristic of classical GDM. To eliminate the potential impact of STZ per se on fetuses and placentas during the modeling process [16], we also retained pregnant mice whose blood glucose levels did not increase following STZ injection (the STZ‐null group) (Figure 1B). Compared to the control (CON) and STZ‐null groups, the fetal crown‐rump length, fetal weight, and placental weight were significantly decreased in the GHG group (Figure 1C–F). Additionally, placental efficiency, defined as the ratio of fetal to placental weight [17], was significantly decreased in the GHG group (Figure 1G), suggesting compromised placental function. However, there was no difference in fetal crown‐rump length, fetal weight, placental weight, and placental efficiency between the CON and STZ‐null groups (Figure 1C–G), eliminating the potential direct impact of STZ on the growth of fetuses and placentas. These results indicate that FGR caused by GHG is associated with placental damage.

### 3.2 Gestational Hyperglycemia Impairs the Fusion and Formation of the Syncytiotrophoblast

**FIGURE 2:**
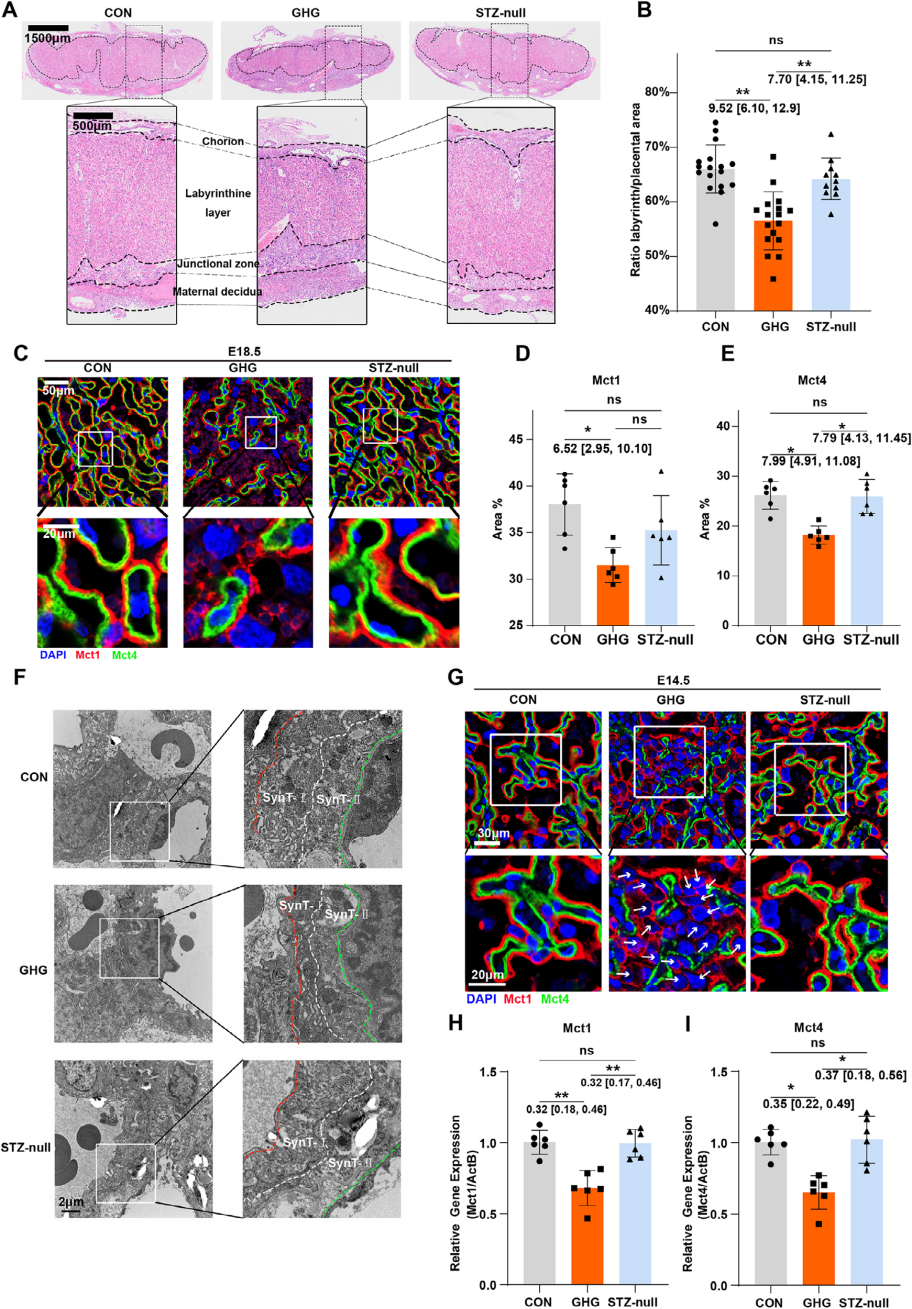
Gestational hyperglycemia impairs fusion and formation of the syncytiotrophoblast. (A) Representative H&E staining of the E18.5 placentas from the CON, GHG, and STZ‐null groups. The dashed outlined region corresponds to the labyrinth zone. (B) The bar plot shows the ratio of labyrinth area to total placental area in the E18.5 placentas (*n* = 11–17 placentas per group). (C) Immunofluorescence for MCT1 (SynT‐I) and MCT4 (SynT‐II) in the E18.5 placentas from the CON, GHG, and STZ‐null groups. (D and E) The ratio of MCT1‐ and MCT4‐positive area in the E18.5 placentas from the CON, GHG, and STZ‐null groups (*n* = 6 individual experiments per group). (F) Representative transmission electron microscopy images of syncytiotrophoblast layers in the E18.5 placentas from the CON, GHG, and STZ‐null groups. Transmission electron microscopy images were captured at a magnification of 3400×. (G) Immunofluorescence for MCT1 (SynT‐I) and MCT4 (SynT‐II) in the E14.5 placentas from the CON, GHG, and STZ‐null groups. Arrows indicate unfused trophoblast cells. (H and I) mRNA levels of *Mct1* and *Mct4* in placentas from the CON, GHG, and STZ‐null groups (*n* = 6 placentas per group). The data are shown as mean ± SD. Multiple placentas from the same dam were considered non‐independent and analyzed using a linear mixed model with dam as a random intercept (B, D, E, H and I). The mean difference between the two groups, along with the 95% confidence interval, is displayed beneath the significance bar. “*n*” indicates the number of independent biological replicates; technical replicates are averaged within each biological replicate before statistical analysis and are not included in the “*n*” values. *p* < 0.05 was considered statistically significant. ^*^ *p* < 0.05; ^**^ *p* < 0.01.

We then further investigated pathological changes in the placenta. Compared to the CON group, the ratio of labyrinth area to total placental area was significantly reduced in the GHG group, indicating that the labyrinth zone is underdeveloped at E18.5 (Figure 2A, B). In the labyrinth zone, two syncytiotrophoblast layers, namely syncytiotrophoblast layer I (SynT‑I) and SynT‐II, are the crucial structures responsible for the exchange of substance and gas [12]. Monocarboxylate transporter 1 (*Mct1)* and *Mct4* serve as marker genes for SynT‐I and SynT‐II, respectively [11]. In the GHG group, the arrangement of the syncytiotrophoblast layers is significantly disordered, and the tight adhesion between the SynT‐I and SynT‐II is disrupted (Figure 2C). Additionally, the proportions and areas of SynT‐I and SynT‐II were both significantly decreased in the GHG group (Figure 2C–F). Meanwhile, GHG leads to an abnormal distribution of maternal‐fetal blood spaces, significantly increasing the maternal blood space while markedly decreasing the fetal blood space (Figure S1A–C), accompanied by significantly reduced perimeters of both maternal and fetal blood spaces (Figure S1A, D, E), indicating a decrease in the feto‐maternal transport surface area. These results suggest that GHG adversely affects syncytiotrophoblast layers and reduces the area of the feto‐maternal exchange interface, thereby impairing fetal growth and development.

The fusion of trophoblast cells is a crucial biological process in the maturation of syncytiotrophoblast [18]. Since the syncytiotrophoblasts mature at E14.5 in mice [19], we evaluated whether trophoblast fusion was affected by GHG. Compared to the CON group, many unfused trophoblast cells were observed in the GHG group at E14.5, accompanied by the impaired formation of the syncytiotrophoblast layers (Figure 2G). Meanwhile, the mRNA expression levels of *Mct1* and *Mct4* were significantly downregulated in the GHG group compared to the CON group (Figure 2H, I). These findings indicate that GHG may impair syncytiotrophoblasts formation by hindering the fusion of trophoblast cells. However, there were no differences in the above‐mentioned parameters of the labyrinth zone and the syncytiotrophoblast between the CON group and the STZ‐null group (Figure 2A–I), which eliminates the potential direct impact of STZ on the syncytiotrophoblast.

### 3.3 TIM1, a Membrane Fusion‐Related Protein, is Decreased in the Placenta by Gestational Hyperglycemia

**FIGURE 3:**
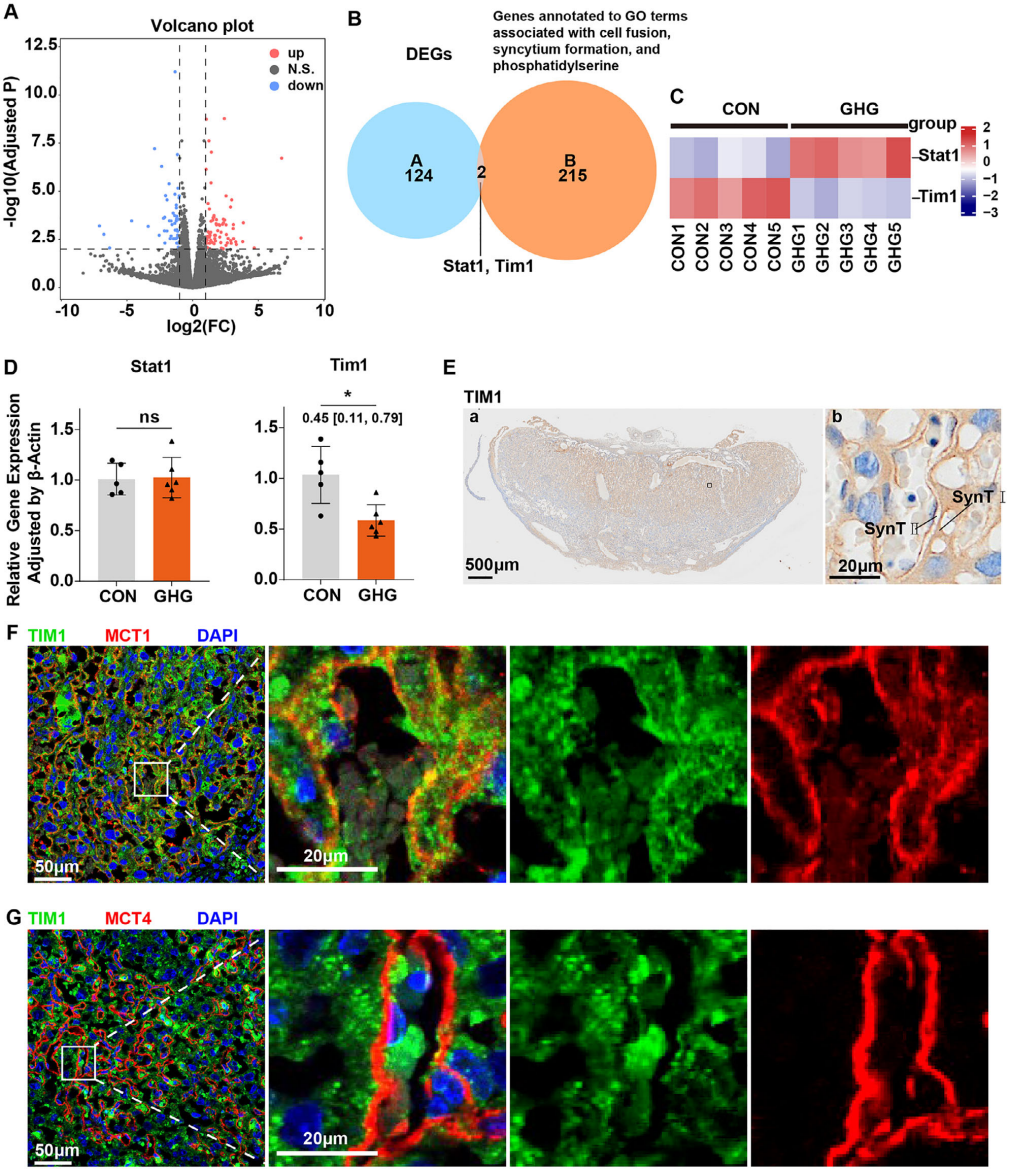
*TIM1* expression is downregulated in the placenta of mice with gestational hyperglycemia. (A) Volcano plot of Differentially Expressed Genes (DEGs) between the placentas from the CON and the GHG groups (*n* = 5 placentas per group). (B) Venn diagram showing the overlap of *Set A* (blue circle) with *Set B* (orange circle). *Set A*: DEGs between the placentas from the CON and the GHG groups. *Set B*: genes contained in GO term associated with cell fusion, syncytium formation and phosphatidylserine. (GO: 0140253, 0034242, 0045026, 0060143, 0060142, 0000768, 0006949, 0061589, 0001786, 0070782, 0140346, 0090556, 0140343, 0017121, 1905780). (C) Heatmap displays the expression trends of genes including *Stat1* and *Tim1* derived from RNA‐seq (*n* = 5 placentas per group). (D) The mRNA expression levels of *Stat1* and *Tim1* in placentas from the CON and GHG groups (*n* = 5–6 placentas per group). (E) Representative TIM1 immunohistochemistry images of E14.5 placentas. (F) Immunofluorescence images for MCT1 (SynT‐I) and TIM1 in E14.5 placentas. (G) Immunofluorescence images for MCT4 (SynT‐II) and TIM1 in E14.5 placentas. Data are shown as mean ± SD. Data were analyzed using unpaired Student's t‐test (D). The mean difference between the two groups, along with the 95% confidence interval, is displayed beneath the significance bar. “*n*” indicates the number of independent biological replicates; technical replicates are averaged within each biological replicate before statistical analysis and are not included in the “*n*” values. *p* < 0.05 was considered statistically significant. ^*^ *p* < 0.01.

Based on the impairment of syncytiotrophoblast formation, we subsequently investigated the underlying mechanisms using RNA sequencing. RNA sequencing results identified 77 significantly upregulated transcripts and 49 downregulated transcripts in the GHG group, while approximately 17 800 genes exhibited comparable levels between control and GHG groups (Figure 3A). To investigate the molecular mechanisms underlying the impaired trophoblast cell fusion by GHG mentioned above, we intersected these differentially expressed genes with gene sets related to cellular fusions. We identified two candidate genes including *Stat1* (signal transducer and activator of transcription 1) and *Tim1* (Figure 3B, C). qPCR validation confirmed decreased expression levels of *Tim1* by GHG, while *Stat1* was not significantly different between the CON and GHG groups (Figure 3D). Moreover, the decreased protein expression level of TIM1 in the GHG placentas was further validated (Figure S2A–D). TIM1 is capable of binding to phosphatidylserine (PS) on the cell membrane, thereby promoting membrane fusion such as viral‐host cell fusion [20, 21]; however, the direct role of TIM1 in cell‐cell fusion remains unknown. Therefore, we assessed the spatial distribution of *TIM1* expression in·the placenta, and found that TIM1 was obviously expressed within the labyrinth zone (Figure 3E). Furthermore, quantitative co‐localization analysis revealed that TIM1 shows substantial but not exclusive overlap with the SynT‐I marker MCT1 (Pearson's R = 0.563; Manders’ M1 = 0.758, M2 = 0.709; Figure 3F; Figure S2E,F), whereas minimal overlap was detected with the SynT‐II marker MCT4 (Pearson's R = −0.009; Manders’ M1 = 0.313, M2 = 0.308; Figure 3G; Figure S2G,H). These results indicate that TIM1 is predominantly associated with SynT‐I cells, with lower‐level presence in other trophoblast subtypes. Together, TIM1, a potential membrane fusion‐related protein, was expressed in the syncytiotrophoblast layer I (SynT‐I) and was downregulated during gestational hyperglycemia.

To clarify the temporal sequence between *Tim1* downregulation and impaired syncytiotrophoblast formation, we examined placentas at earlier gestational stages. As the mouse placenta is not clearly differentiated at E8.5 [22, 23, 24], we included sampled collected at E10.5 for further analysis. *Tim1* downregulation in the GHG group was already evident at E10.5 (Figure S3A–C), while no significant differences in trophoblast cell fusion were observed between the two groups at this stage (Figure S3D,E). These observations suggest that *Tim1* downregulation occurs prior to evident syncytiotrophoblast deformation.

### 3.4 TIM1 Promotes the Fusion of Trophoblast Cells

**FIGURE 4:**
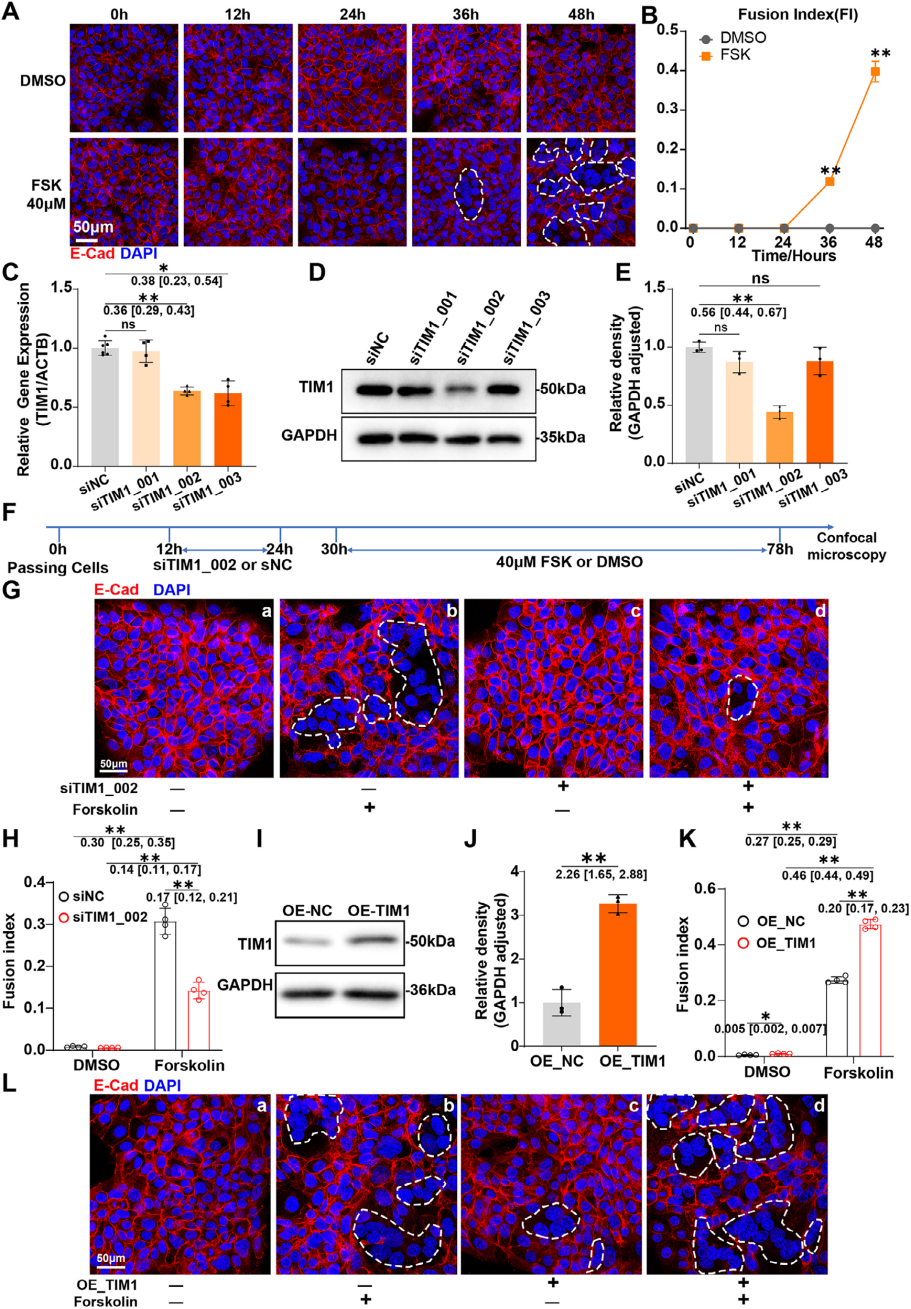
TIM1 promotes the fusion of trophoblast cells. (A) Representative images of the 40 µm FSK‐induced syncytium formation in BeWo cells, with the white dotted lines indicating multinucleated cells. E‐Cadherin (E‐Cad) delineates BeWo trophoblast boundaries (red). DAPI labels nuclei (blue). (B) Quantification of the fusion index in 40 µm FSK‐ or DMSO‐treated BeWo cells (*n* = 3 independent experiments per group). (C) Effects of *TIM1*‐specific siRNAs (siTIM1_001, 002, 003) on the mRNA expression of *TIM1* in BeWo cells (*n* = 4–6 independent experiments per group). siNC: nontargeting negative control siRNAs, used to exclude non‐specific effects. (D‐E) Effects of *TIM1*‐specific siRNAs (siTIM1_001, 002, 003) on the protein expression of *TIM1* in BeWo cells (*n* = 3 independent experiments per group). (F) Schematic diagram of the experimental design: After passaging, BeWo cells were treated with siTIM1_002 to knock down *TIM1*, followed by stimulation with 40 µm FSK. (G) Representative immunostaining of E‐Cad (red) and DAPI (blue) in BeWo cells, with the white dotted lines indicating multinucleated cells. (H) Quantification of the fusion index in BeWo cells (*n* = 4 independent experiments per group). (I) Representative Western blot image of TIM1 protein expression. (J) Quantification of TIM1 protein expression efficiency in TIM1‐overexpressed BeWo cells (*n* = 3 independent experiments per group). (K) Quantification of the fusion index in BeWo cells (*n* = 4 independent experiments per group). (L) Representative immunostaining of E‐Cad (red) and DAPI (blue) in BeWo cells, with the white dotted lines indicating multinucleated cells. The data are shown as mean ± SD. Data were square root–transformed prior to statistical analysis (H,K). Data were analyzed using unpaired Student's *t*‐test (B and J), Welch ANOVA followed by Games–Howell post hoc analysis (C), one‐way ANOVA followed by Tukey post hoc analysis (E), and two‐way ANOVA followed by Tukey post hoc analysis (H and K). The mean difference between the two groups, along with the 95% confidence interval, is displayed beneath the significance bar. “*n*” indicates the number of independent biological replicates; technical replicates are averaged within each biological replicate before statistical analysis and are not included in the “*n*” values. *p* < 0.05 was considered statistically significant. ^*^ *p* < 0.01, ^**^ *p* < 0.001.

We hypothesized that the downregulated TIM1 may contribute to trophoblast cell fusion dysfunction in GHG. To verify this hypothesis, we further explored the role of TIM1 on trophoblast cell fusion using a forskolin (FSK)‐induced BeWo cell fusion model [25]. After 48 h of stimulation with FSK (40 µm), cell fusion and noticeable syncytium formation was observed (Figure 4A,B). Subsequently, we designed three siRNAs targeting *TIM1*. siTIM1_002 significantly inhibited the mRNA and protein expression of TIM1 in BeWo cells (Figure 4C–E). We then knocked down TIM1 in BeWo cells, followed by stimulation with FSK (Figure 4F). In the absence of FSK stimulation, TIM1 knockdown had no significant effect on BeWo cells (Figure 4G‐a,c, H). However, TIM1 silence significantly inhibited FSK‐induced fusion of BeWo cells (Figure 4G‐d, H). Additionally, we conducted overexpression of TIM1 in BeWo cells (Figure 4I, J), and assessed its effects on BeWo cell fusion. Compared to control lentiviral transfection, TIM1 overexpression elevated the fusion index of FSK‐induced BeWo cells (Figure 4L, K). These results suggest that TIM1 promotes trophoblast fusion in a widely used in vitro model, and suggest that TIM1 downregulation underlies the trophoblast fusion defects observed in GHG‐induced FGR.

### 3.5 *Tim1* Deficiency Results in Impaired Syncytiotrophoblast Formation and Fetal Growth Restriction In Vivo

**FIGURE 5:**
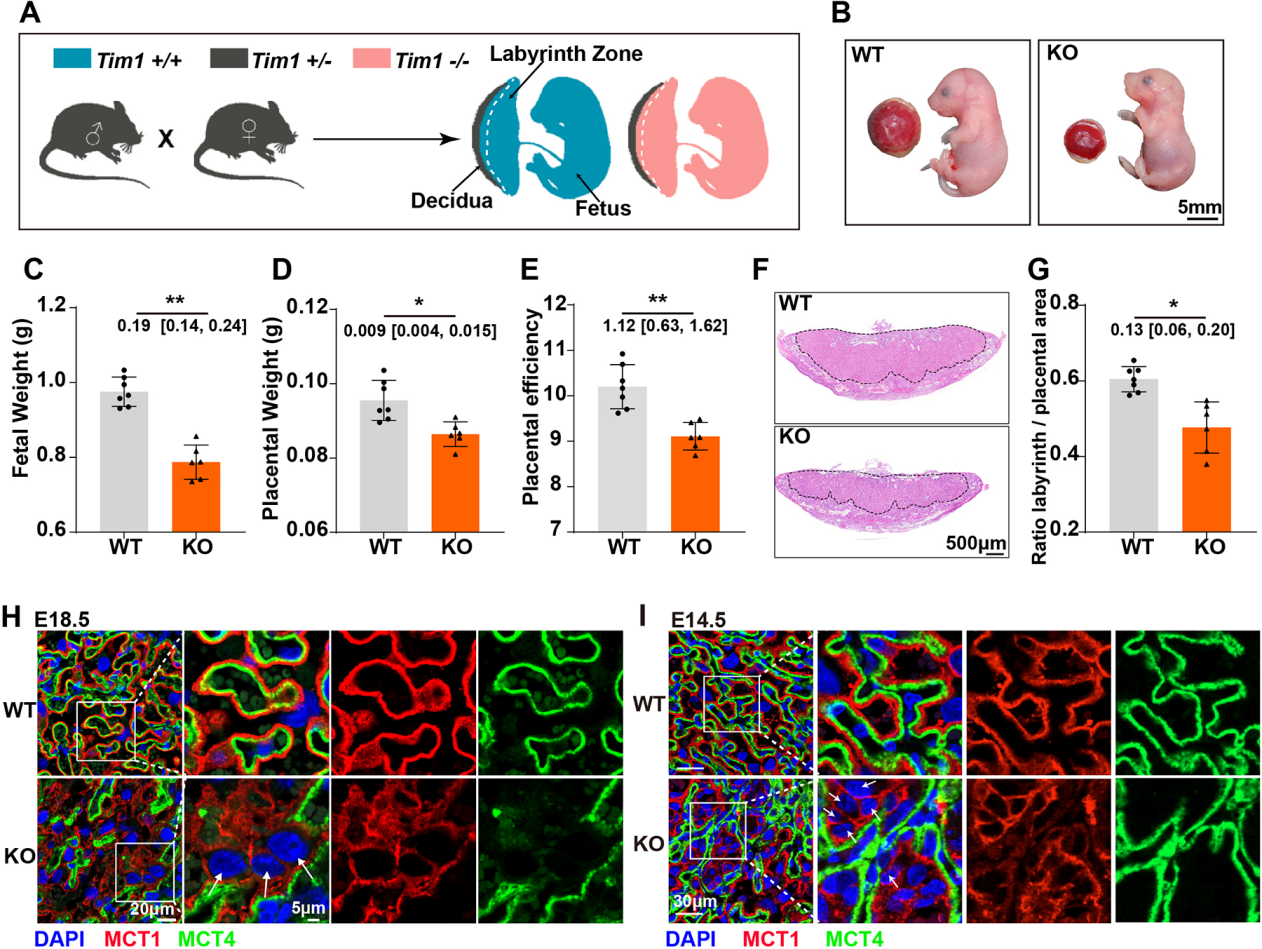
*Tim1* KO mice exhibit impaired syncytiotrophoblast formation and fetal growth restriction. (A) Breeding scheme diagram: By mating HET (*Tim1^+/–^*) × HET, obtain WT (*Tim1* ^+/+^) and KO (*Tim1* ^−/−^) fetuses and placentas. (B) Representative images of fetuses and placentas from WT and KO mice at E18.5. (C–E) Bar plots showing placental weights (C), fetal weights (D), and placental efficiency (E) from WT and KO groups at E18.5 (*n* = 6–7 per group). (F) Representative H&E staining images of placentas in WT and KO groups at E18.5. The dashed outlined region corresponds to the placental labyrinth zone. (G) Bar plot showing the ratio of labyrinth area to total placental area in WT and KO groups at E18.5 (*n* = 6–7 placentas per group). (H) Immunofluorescence for MCT1 and MCT4 in the E18.5 placentas from WT and KO groups. Arrows indicate unfused trophoblast cells. (I) Immunofluorescence for MCT1 and MCT4 in E14.5 placentas from WT and KO groups. Arrows indicate unfused trophoblast cells. Data are presented as mean ± SD. Multiple fetuses or placentas from the same dam were considered non‐independent and analyzed using a linear mixed model with dam as a random intercept (C, D, E, G). The mean difference between the two groups, along with the 95% confidence interval, is displayed beneath the significance bar. “*n*” indicates the number of independent biological replicates; technical replicates are averaged within each biological replicate before statistical analysis and are not included in the “*n*” values. *p* < 0.05 was considered statistically significant. ^*^ *p* < 0.01, ^**^ *p* < 0.001.

Based on the cellular findings, we further explored the role of *Tim1* in trophoblast physiology and placental development in vivo. We first generated a *Tim1*‐deficient mouse strain (Figure S4A–C). We employed a *Tim1* ^+/−^× *Tim1* ^+/−^ (HET) breeding scheme to obtain fetuses with *Tim1* ^+/+^ (WT) and *Tim1* ^−/−^ (KO) genotypes for subsequent experimental analyses, and *Tim1* deficiency did not alter litter size (Figure S4E). Given that all dams were HET, the genotypes of maternal decidua in both WT and KO placentas were uniformly HET (Figure 5A). Compared with WT placentas from WT dams, KO placentas from HET dams exhibited a loss of TIM1 expression in the labyrinth zone and a slight reduction of TIM1 expression in the maternal decidua (Figure S4D). This further reflects that *Tim1* was successfully knocked out in the labyrinth zone.

We then first examined the phenotypes of WT and KO fetuses and placentas from HET dams at E18.5. Compared with the WT group, *Tim1* KO resulted in reduced fetal weight, placental weight, and placental efficiency (Figure 5B–E). In addition, in *Tim1* KO placentas, the proportion of the labyrinth zone was reduced (Figure 5F,G), and the formation of syncytiotrophoblasts was impaired, exhibiting morphological abnormalities (Figure 5H). To investigate whether trophoblast fusion was hindered, we further analyzed placentas at E14.5. Notably, the formation of the syncytiotrophoblast layer was impeded in *Tim1* KO placentas, with increased unfused trophoblast cells (Figure 5I) and a decreased proportion of the labyrinth zone but not the decidual zone (Figure S5A–D). These findings indicate that *Tim1* KO impairs syncytiotrophoblast formation by inhibiting trophoblast fusion, potentially contributing to FGR.

### 3.6 Hyperglycemia Inhibits Trophoblast Cell Fusion Through ROS‐Induced Downregulation of *TIM1*

**FIGURE 6:**
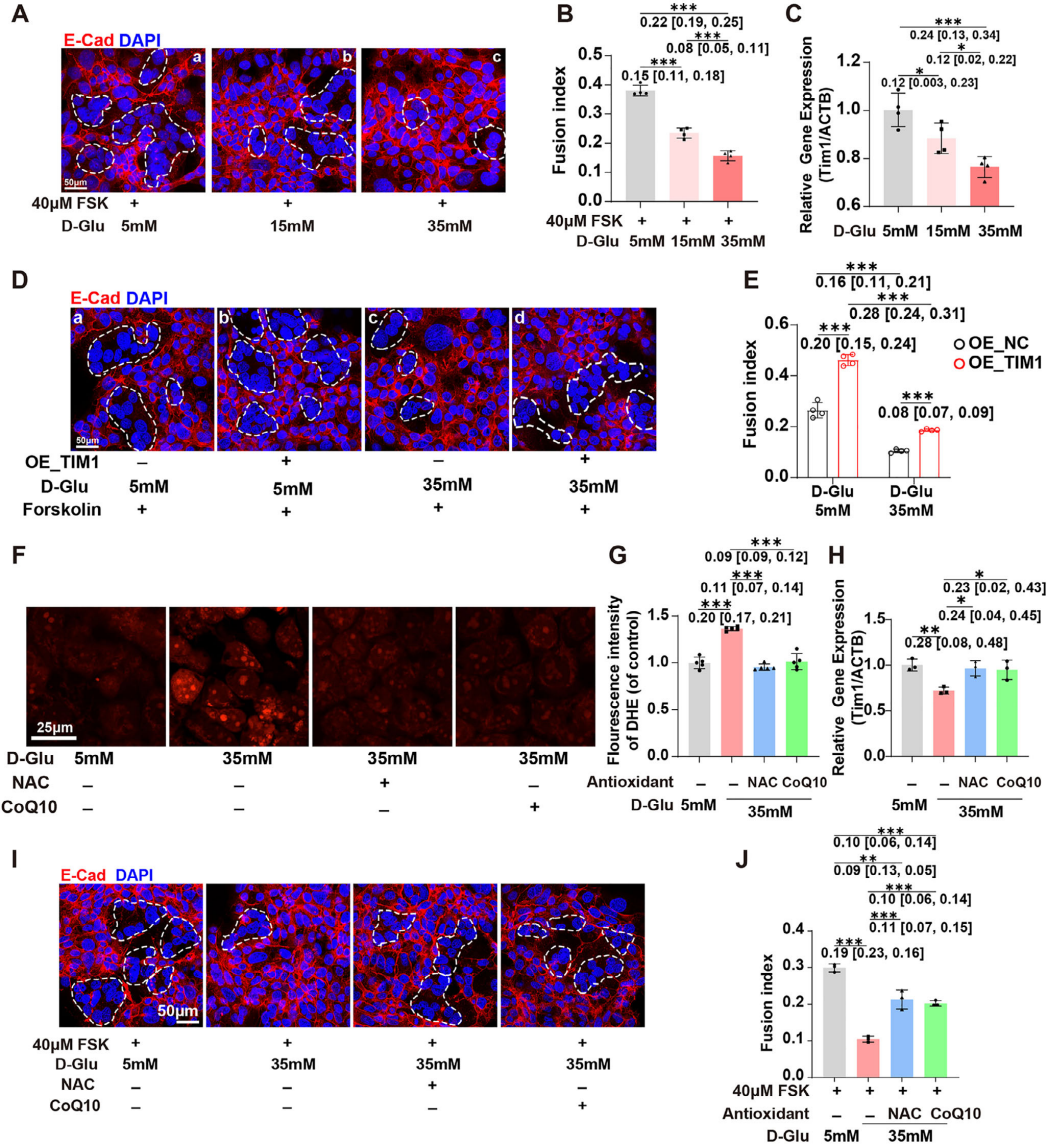
High glucose inhibits trophoblast cell fusion through ROS‐induced downregulation of *TIM1*. (A) Representative immunostaining of E‐Cadherin (E‐Cad; red) and DAPI (blue) in BeWo cells treated with D‐Glucose (D‐Glu; 5, 15, 35 mm). The white dotted lines indicate multinucleated cells, serving as indicators of trophoblast fusion levels. (B) Quantification of the fusion index in BeWo cells treated with D‐Glu (5, 15, 35 mm) (*n* = 4 independent experiments per group). (C) *TIM1* mRNA expression in BeWo cells treated with D‐Glu (5, 15, 35 mm) (*n* = 4 independent experiments per group). (D) Representative immunostaining of E‐Cad (red) and DAPI (blue) in BeWo cells treated with D‐Glu (5, 35 mm), with or without *TIM1* overexpression. The white dotted lines indicate multinucleated cells. (E) Quantification of the fusion index in BeWo cells treated with D‐Glu (5, 35 mm), with or without *TIM1* overexpression (*n* = 4 independent experiments per group). (F) Dihydroethidium (DHE) staining to measure steady‐state ROS levels. (G) Quantification of DHE fluorescence intensity (*n* = 5 independent experiments per group). (H) *TIM1* mRNA expression in BeWo cells treated with NAC (5 mm) or CoQ10 (10 µm) under high‐glucose (35 mm D‐Glu) conditions (*n* = 3 independent experiments per group). (I) Representative immunostaining of E‐Cad (red) and DAPI (blue) in BeWo cells treated with D‐Glu (5 mm), D‐Glu (35 mm), D‐Glu (35 mm) with NAC, and D‐Glu (35 mm) with CoQ10. The white dotted lines indicate multinucleated cells. (J) Quantification of the fusion index in BeWo cells treated with D‐Glu (5 mm), D‐Glu (35 mm), D‐Glu (35 mm) with NAC, and D‐Glu (35 mm) with CoQ10 (*n* = 3 independent experiments per group). Data expressed as mean ± SD. Data were analyzed using one‐way ANOVA followed by Tukey post hoc analysis (B, C, G, H and J) and two‐way ANOVA followed by Tukey post hoc analysis (E). The mean difference between the two groups, along with the 95% confidence interval, is displayed beneath the significance bar. “*n*” indicates the number of independent biological replicates; technical replicates are averaged within each biological replicate before statistical analysis and are not included in the “*n*” values. *p* < 0.05 was considered statistically significant. ^*^ *p* < 0.05; ^**^ *p* < 0.01; ^***^ *p* < 0.001.

Based on the pivotal role of *TIM1* in trophoblast cell fusion, we next explored whether it mediated hyperglycemia‐induced trophoblast cell fusion defect. We first verified the impact of high glucose stimulation on BeWo cell fusion. We found that D‐Glu dose‐dependently decreased FSK‐induced fusion of BeWo cells (Figure 6A, B), accompanied by a concomitant reduction in *TIM1* mRNA expression (Figure 6C). To demonstrate whether hyperglycemia inhibited BeWo cell fusion by reducing *TIM1* expression, we conducted *TIM1* overexpression in BeWo cells. Compared to control vector transfection, *TIM1* overexpression partially but significantly rescued the inhibition of BeWo cell fusion induced by high D‐Glu (Figure 6D, E). These results suggest that downregulation of *TIM1* contributes to hyperglycemia‐induced trophoblast cell fusion defect.

We further investigated the mechanism by which high glucose suppresses *TIM1* expression. Given that diabetes induces chronic oxidative stress in various organs including the placenta [26, 27, 28], we further evaluated the levels of reactive oxygen species (ROS) in BeWo cells treated with high glucose. We observed that high glucose elevated ROS levels in BeWo cells (Figure 6F,G) while ROS clearance by antioxidants (N‐Acetylcysteine, NAC or Coenzyme Q10, CoQ10), two effective antioxidants [29], significantly reversed *TIM1* expression levels in high glucose‐treated cells (Figure 6H), suggesting that ROS might account for decreased *TIM1* expression. We thus further directly induced oxidative stress by treatment with H_2_O_2_ in vitro. H_2_O_2_ dose‐dependently decreased *TIM1* mRNA expression levels (Figure S6A), which were reversed by NAC or CoQ10 (Figure S6B). These findings suggest that oxidative stress directly mediates the downregulation of *TIM1* expression.

We then explored the protective effects of anti‐oxidative treatment on cell fusion defects induced by high glucose in vitro. Treatment with NAC or CoQ10 both significantly improved fusion index in high glucose‐treated BeWo cells (Figure 6I,J). To establish whether the protective effect of antioxidants is mediated by *TIM1*, we silenced *TIM1* under high glucose culture conditions and examined whether antioxidants could still rescue the impaired cell fusion. Under control siRNA transfection, CoQ10 significantly improved cell fusion; however, this protective effect was eliminated when *TIM1* is silenced, demonstrating a critical role of *TIM1* in mediating CoQ10‐exerted protection against cell fusion impairment (Figure S6C,D). We also excluded the possibility that *TIM1* directly regulates ROS contents, as *TIM1* overexpression alone under normal or high‐glucose conditions did not affect ROS levels in BeWo cells (Figure S6E,F). Together, hyperglycemia inhibits trophoblast cell fusion through ROS‐induced downregulation of *TIM1*, and anti‐oxidative treatments exert a protective effect.

### 3.7 Antioxidant Therapy Alleviates Hyperglycemia‐Induced Syncytiotrophoblast Formation Defect and Fetal Growth Restriction In Vivo

**FIGURE 7:**
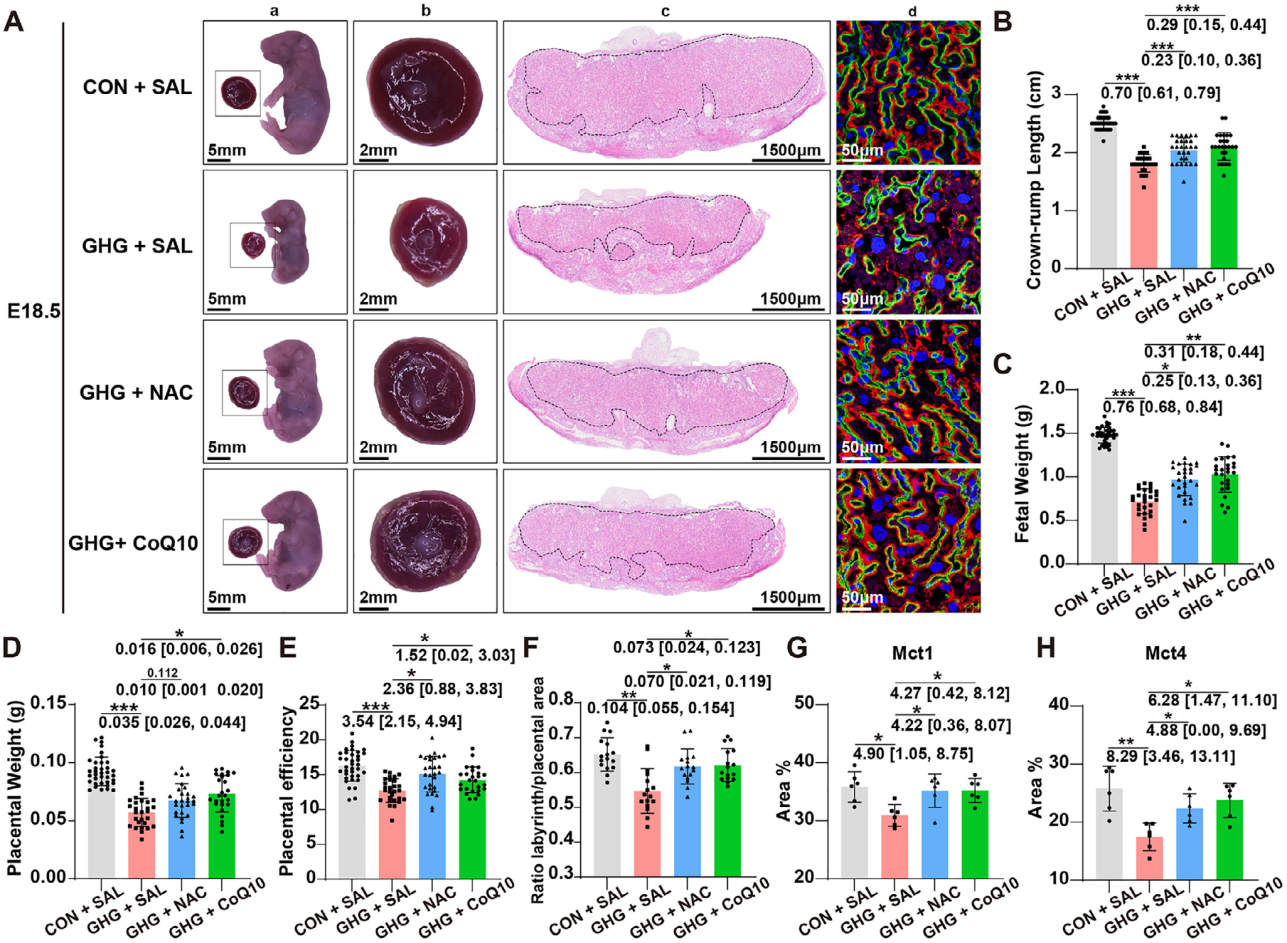
Antioxidant therapy alleviates hyperglycemia‐induced impairment of syncytiotrophoblast formation and fetal growth restriction. (A) Comparison of the gross characteristics of fetuses and placentas, representative H&E staining of placentas, and immunofluorescence for MCT1 (SynT‐I) and MCT4 (SynT‐II) from the CON+SAL, GHG+SAL, GHG+NAC, and GHG+CoQ10 groups at E18.5. (B) Crown‐rump length of E18.5 fetuses from the CON+SAL, GHG+SAL, GHG+NAC, and GHG+CoQ10 groups (*n* = 26–36 fetuses per group). (C) Fetal weight of E18.5 fetuses from the CON+SAL, GHG+SAL, GHG+NAC, and GHG+CoQ10 groups (*n* = 26–36 fetuses per group). (D) Placental weight of the E18.5 placentas from the CON+SAL, GHG+SAL, GHG+NAC, and GHG+CoQ10 groups (*n* = 26–36 placentas per group). (E) Placental efficiency of the E18.5 placentas from the CON+SAL, GHG+SAL, GHG+NAC, and GHG+CoQ10 groups (*n* = 26–36 placentas per group). (F) Ratio of labyrinth area to total placental area in the E18.5 placentas from the CON+SAL, GHG+SAL, GHG+NAC, and GHG+CoQ10 groups (*n* = 16 placentas per group). (G and H) Ratio of MCT1‐ and MCT4‐positive areas in the E18.5 placentas from the CON+SAL, GHG+SAL, GHG+NAC, and GHG+CoQ10 groups (*n* = 6 placentas per group). Data are presented as mean ± SD. Multiple fetuses or placentas from the same dam were considered non‐independent and analyzed using a linear mixed model with dam as a random intercept (B–H). The mean difference between the two groups, along with the 95% confidence interval, is displayed beneath the significance bar. “*n*” indicates the number of independent biological replicates; technical replicates are averaged within each biological replicate before statistical analysis and are not included in the “*n*” values. *p* < 0.05 was considered statistically significant. ^*^ *p* < 0.05; ^**^ *p* < 0.01; ^***^ *p* < 0.001.

Building upon the in vitro findings, we then treated GHG mice with antioxidants during pregnancy. GHG significantly downregulated TIM1 expression in the placental labyrinth zone, whereas NAC and CoQ10 effectively restored TIM1 expression (Figure S7A,B). In addition, NAC and CoQ10 increased fetal crown‐rump length and fetal weight in mice affected by GHG (Figure 7A–C). Additionally, NAC and CoQ10 mitigated the reduction in placental weight and improved placental efficiency caused by GHG (Figure 7A, D, E). Furthermore, both NAC and CoQ10 partially restored the proportion of the labyrinth area in the GHG placenta (Figure 7A, F), and increased the proportions of the two syncytiotrophoblast layers, SynT‐I (MCT1+) and SynT‐II (MCT4+), in the GHG placenta (Figure 7A, G, H). These findings raise the possibility that antioxidant therapy may help ameliorate placental defects and FGR associated with hyperglycemia during pregnancy; however, this observation is hypothesis‐generating and warrants further investigation.

### 3.8 The Expression of *TIM1* is Decreased in Human Placentas Exposed to Hyperglycemia During Pregnancy

**FIGURE 8:**
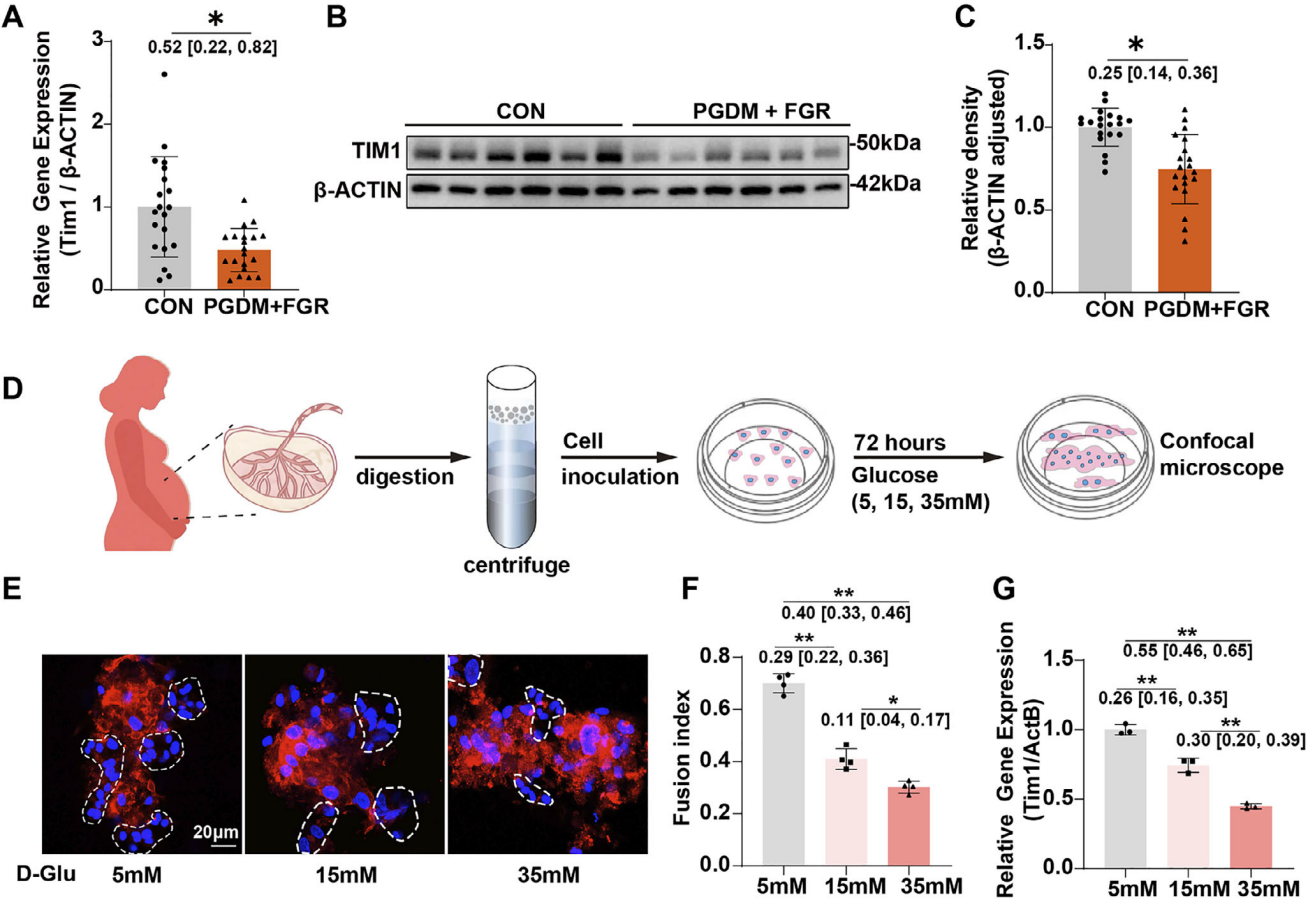
*TIM1* expression is decreased in the placenta affected by pre‐gestational diabetes and fetal growth restriction. (A) mRNA expression levels of *TIM1* in placentas (*n* = 20 placentas per group). (B) Representative Western blot image showing TIM1 protein expression in placentas. (C) Quantification of TIM1 protein expression in placentas (*n* = 20 placentas per group). (D) Schematic representation of the isolation of primary human trophoblast cells followed by in vitro culture and treatment. (E) Primary human trophoblast cells fuse to form syncytia in culture media containing 5, 15, or 35 mm D‐Glu. E‐Cadherin (E‐Cad, red) and DAPI (blue). (F) Quantification of the fusion index in primary human trophoblast cells treated with D‐Glu (5, 15, or 35 mm) (*n* = 4 independent experiments per group). (G) *TIM1* mRNA expression in primary human trophoblast cells treated with D‐Glu (5, 15, 35 mm) (*n* = 3 independent experiments per group). Data are presented as mean ± SD. Statistical analysis was performed using multiple linear regression models with BMI included as a covariate. Model assumptions were assessed by examining residual normality and homogeneity of regression slopes (A and C). Data were analyzed using one‐way ANOVA followed by Tukey post hoc analysis (F and G). The mean difference between the two groups, along with the 95% confidence interval, is displayed beneath the significance bar. “*n*” indicates the number of independent biological replicates; technical replicates are averaged within each biological replicate before statistical analysis and are not included in the “*n*” values. *p* < 0.05 was considered statistically significant. ^*^ *p* < 0.01, ^**^ *p* < 0.001.

To explore the clinical relevance of the findings in mice, we recruited control pregnant participants and women with pre‐gestational diabetes (PGDM) and FGR (*n* = 20), and collected placental tissue samples. Table S1 summarizes the baseline characteristics of all participants. Women with PGDM and FGR had higher pre‐pregnancy body mass index (BMI), early pregnancy glycated hemoglobin (HbA1c), and fasting blood glucose levels compared to the control group (Table S1). Consistent with findings from animal experiments, the mRNA (Figure 8A) and protein (Figure 8B, C) levels of TIM1 were significantly reduced in the placental tissue of women with PGDM and FGR. Correlation analysis revealed that elevated maternal fasting blood glucose levels were associated with reduced placental *TIM1* expression, while decreased placental *TIM1* expression was associated with lower birth weight (Figure S8A,B). To further validate the direct impact of hyperglycemia on human placental trophoblasts, primary human trophoblasts were isolated from term placentas and cultured for 72 h in media containing 5, 15, or 35 mm glucose (Figure 8D). Glucose concentrations dose‐dependently inhibited cell fusion (Figure 8E, F) and reduced *TIM1* mRNA expression in primary human trophoblasts (Figure 8G).

## 4 Discussion

In the present study, we elucidated novel insights into the mechanisms underlying GHG related FGR. First, GHG‐induced FGR was associated with impaired placental efficiency, which is attributable to defective trophoblast cell fusion and syncytiotrophoblast formation in the placenta. Second, gene expression profiling and staining analysis of the placenta revealed that *Tim1* was expressed in the syncytiotrophoblast, and was downregulated in the GHG placenta. *Tim1* downregulation leads to defective syncytiotrophoblast formation by inhibiting trophoblast cell fusion. Third, hyperglycemia downregulated *Tim1* expression in trophoblasts through increased ROS, and antioxidant treatment improved defective formation of syncytiotrophoblasts and FGR in in vitro and in vivo models. Fourth, the reduction of *TIM1* expression was confirmed in the human placentas with gestational hyperglycemia and FGR, and inhibition of cell fusion by hyperglycemia was confirmed in primary human trophoblasts. Collectively, the current study provides the first evidence that gestational hyperglycemia causes FGR at least partly through defective syncytiotrophoblast formation and reduced placental efficiency, and identifies TIM1 as a novel regulator of trophoblast cell fusion to form syncytiotrophoblast during pregnancy.

Diabetes and other metabolic disorders are becoming increasingly prevalent and pose critical health challenges [30, 31]. Of note, diabetes existing from the early stages of pregnancy increases the risk of FGR [5, 6], which is frequently recapitulated in animal models of diabetic pregnancies [32]. Several mechanisms underlying GHG‐related FGR have been reported, such as maternal microangiopathy and elevated levels of inflammatory burdens [33, 34]. Previous studies have confirmed that immune alterations are closely associated with hyperglycemia‐induced FGR. For instance, in STZ‐induced diabetic mouse models, elevated levels of cytotoxic natural killer (NK) cells in the placenta have been observed, which are strongly correlated with FGR [35]. However, the mechanisms underlying GHG‐related FGR remain incompletely understood. Current study observed that GHG‐induced FGR was associated with decreased placental efficiency and defective formation of syncytiotrophoblast. The syncytiotrophoblast is a critical structure for maternal‐fetal material exchange [10], and essential for fetal growth and development. Severe syncytiotrophoblast defects in mice have been reported to cause congenital heart disease [14] and even fetal death [13]. Therefore, current findings reveal a new pathological mechanism of GHG‐induced FGR involving deformation of syncytiotrophoblasts, which provides a complementary perspective to previously identified immune‐related mechanisms.

Cell‐cell fusion is a fundamental cellular process necessary for many biological processes including syncytiotrophoblast formation during pregnancy [36]. The syncytiotrophoblast is formed through the differentiation and fusion of trophoblasts [37]. This process depends on delicately regulated molecular signalings [36, 38, 39]. For example, syncytins bridge the plasma membrane and directly merge trophoblast cells into multinucleated structures by binding to their specific receptors, such as solute carrier family 1 member 5 (SLC1A5) and major facilitator superfamily domain containing 2 (MFSD2A) [18, 37]. In addition, phosphatidylserine (PS)‐dependent pathways also implicate in trophoblast fusion. Deficiency of transmembrane protein 16 (TMEM16F), a factor responsible for membrane exposure of PS, inhibits trophoblast fusion and the formation of syncytiotrophoblast [40]. In this study, we observed that trophoblast fusion was impaired by GHG in mouse models. Although prior human studies have not directly linked gestational diabetes with FGR to impaired trophoblast fusion, they have—consistent with our findings in mice—reported placental and trophoblast abnormalities. Previous studies have shown that hyperglycemia during pregnancy induces various pathological changes in the placenta, such as increased syncytial knots, stromal edema, villous thrombosis, and calcification [4, 41, 42, 43]. Specifically, in pregnancies complicated by FGR, placentas exhibit an even greater number of syncytial knots in the syncytiotrophoblast, suggesting that the presence of placental dysfunction in human samples [44, 45]. Here, we further observed that cell fusion was also impaired by hyperglycemia in primary human trophoblasts. Moreover, we identified TIM1 as a novel regulator of trophoblast fusion. We observed that *TIM1* is expressed in syncytiotrophoblast layer I and is downregulated in GHG. *TIM1* belongs to the *TIM* family. The *TIM* family encodes type‐I membrane proteins, including *TIM1*, *TIM3*, and *TIM4*, which play key roles in regulating multiple pathological processes, including allergies, transplant tolerance, and responses to viral infections [46]. *TIM1* was previously reported to promote kidney repair after acute injury [47] and regulate tumor growth [48]. Of interest, TIM1 specifically recognizes PS exposed on the cell surface [46]. By interacting with PS, TIM1 promotes the fusion of the viral envelope with the host cell membrane, thereby facilitating viral fusion [20]. This suggests that TIM1 may be involved in regulating cell‐to‐cell fusion, albeit the lack of report in this regard. Here, we found that TIM1 promoted trophoblast fusion, and reduced *TIM1* expression contributed to the inhibitory effects of high glucose stimulation on trophoblast fusion. Additionally, we observed that *Tim1* deficiency alone mimicked the phenotypes induced by GHG in mice, including impaired syncytiotrophoblast formation, decreased placental weight and fetal weight. A further investigation revealed that downregulation of *TIM1* was caused by hyperglycemia‐induced oxidative stress, and antioxidants administration reversed *TIM1* expression, and significantly improved FGR and defective syncytiotrophoblast formation in GHG mouse model. However, the regulatory mechanisms mediating oxidative stress–induced *Tim1* downregulation remain unclear. Gene expression is governed by both transcriptional and post‐transcriptional regulatory layers. Notably, transcription factors (TFs) such as NRF2 are highly responsive to oxidative stress [49], and activation of NRF2 has been reported to suppress *Tim1* expression in renal epithelial cells [50]. Although this evidence was obtained in a kidney context, it suggests a conserved stress‐responsive regulatory axis that may also be relevant in placental trophoblasts under hyperglycemic oxidative stress. Beyond transcriptional regulation, *Tim1* can be post‐transcriptionally targeted by microRNAs such as miR‐142‐3p [51], which has been reported to be upregulated in response to oxidative stress [52]. Thus, oxidative stress–induced *Tim1* downregulation in the GHG model may involve coordinated transcriptional repression by oxidative stress–responsive TFs and post‐transcriptional suppression mediated by stress‐induced microRNAs. These regulatory layers position *Tim1* within broader oxidative stress–responsive networks and represent testable hypotheses for future mechanistic investigations. Together, our findings reveal TIM1 as a novel regulator of trophoblast fusion, whose downregulation impairs syncytiotrophoblast formation and contributes to FGR.

This study has several limitations. First, STZ was used to induce maternal diabetes in our model. Although the suppressed placental vascularization observed in our study is consistent with previous reports [53, 54], placental weight has been reported to remain unchanged or even increased in STZ‐induced diabetic rats [55, 56]. Therefore, concerns may arise regarding the representativeness of the current model. However, our study employed a mouse model, which exhibits more consistent placental phenotypes compared with rats [57, 58] and closely recapitulates the features of early gestational hyperglycemia–induced FGR observed in humans [5, 6]. Second, STZ‐induced hyperglycemia models may introduce bias to the conclusions due to the direct effects of STZ, which was at least partially excluded by utilizing STZ‐null mice (in which hyperglycemia was not induced by STZ) as a control. Third, the STZ model effectively mimics severe and persistent gestational hyperglycemia occurring early in pregnancy, representing a metabolic profile more consistent with PGDM. Therefore, extrapolation of the present findings to mild or late‐onset gestational hyperglycemia characteristic of the classical GDM should be made with caution, as the STZ model does not capture mild late‐onset GDM. Importantly, although the magnitude of hyperglycemia differs between PGDM‐like and mild GDM models, previous studies using diet‐induced or moderate gestational hyperglycemia models have reported comparable placental structural and trophoblast differentiation defects, including impaired development of the placental labyrinth [59]. Whether specific phenotypes involving *Tim1* deficiency, disrupted trophoblast fusion, and syncytiotrophoblast deformation are conserved across these models warrants further investigation. Fourth, although we confirmed the downregulation of TIM1 in placentas from humans with PGDM and FGR, the limited number of samples may constrain the external validity of the findings, indicating that the results may not be generalizable to all populations or clinical settings. Therefore, these human data should be interpreted with caution. Fifth, we observed impaired trophoblast cell fusion in the placenta of *Tim1* ^−/−^ mice, accompanied by a reduction—but not a complete absence—of syncytiotrophoblast layer I formation. This finding suggests the involvement of additional mechanisms influencing trophoblast cell fusion, which warrants further investigation. Sixth, the precise upstream transcriptional regulators responsible for oxidative stress‐induced *Tim1* downregulation were not experimentally identified in this study and remain to be elucidated in future work. Seventh, fetal and placental sex were not determined in the present study. Therefore, a potential influence of sex on the placental response to gestational hyperglycemia cannot be excluded. This constitutes another limitation of the study, and future research is required to assess sex‐dependent effects. Finally, some of our analyses based on bulk placental tissue may overlook cell‐specific or spatial‐specific signals, potentially resulting in the omission of critical alterations. In the future, single‐cell or spatial omics technologies may facilitate the identification of novel cell‐ or region‐specific mechanisms. Despite these limitations, our findings provide mechanistic insights with direct relevance to potential clinical interventions.

Our findings are of important clinical significance for the management of FGR, particularly in the context of gestational hyperglycemia. The observed association between gestational hyperglycemia and FGR underscores the necessity of intensified blood glucose monitoring and strict glycemic control after conception, with the aim of reducing the detrimental effects of hyperglycemia on placental function and fetal development. Current preclinical findings raise the hypothesis that CoQ10 may improve placental function and promote fetal growth in gestational hyperglycemia; however, its mechanistic efficacy in humans has not yet been established. Future clinical trials will be essential to evaluate its efficacy in treating diabetes‐related FGR, offering a new therapeutic approach for managing diabetic pregnancies. Additionally, research on small molecules or biologics targeting TIM1, a key player involved in trophoblast fusion identified in current study, could provide new therapeutic options for FGR caused by gestational hyperglycemia.

In summary, we demonstrated defective syncytiotrophoblast formation as a novel pathological mechanism contributing to GHG‐induced FGR, which is attributed to downregulated *TIM1* expression and impaired trophoblast fusion. Moreover, GHG inhibited *TIM1* expression via increased intrauterine ROS, and antioxidant treatment may have potential in mitigating hyperglycemia‐induced syncytiotrophoblast abnormality and FGR, although this remains to be further investigated.

## Author Contributions

J.S., R.L., and C.F. contributed equally to this work. J.S. and designed and performed experiments and analyzed data. J.S. and L.G. wrote and edited the manuscript. J.S., R.L., C.F., S.Z. and L.G. contributed to the study design, conducted experiments, and assisted with data analysis. F.G., Y.L., M.D., and H.W. contributed to the discussion and edited the manuscript. B.Z., Y.H., Z.Y. and J.S.Z contributed to the study design and discussion and edited the manuscript. L.G., H.H. and J.S.Z designed and supervised the research, contributed to the discussion, and edited the manuscript. H.H. and L.G.are the guarantors of this work and, as such, had full access to all data in the study and take responsibility for the integrity and accuracy of the data analysis.

## Funding

Current study was supported by National Key Research and Development Program of China (2022YFC2703000, 2022YFC2703800), National Natural Science Foundation of China (82088102, 82171613, 82471732, 82071731, 81601238), Chinese Academy of Medical Sciences Innovation Fund for Medical Sciences (2019‐I2M‐5‐064), Zhejiang Province College Student Science and Technology Innovation Activity Program (2025R401B184), the Science and Technology Commission of Shanghai Municipality (21Y11907600), Shanghai Municipal Commission of Health and family planning (20215Y0216), Collaborative Innovation Program of Shanghai Municipal Health Commission (2020CXJQ01), Clinical Research Plan of Shanghai Hospital Development Center (SHDC2020CR1008A), Shanghai Clinical Research Center for Gynecological Diseases (22MC1940200), Shanghai Urogenital System Diseases Research Center (2022ZZ01012), Shanghai Frontiers Science Research Base of Reproduction and Development.

## Conflicts of Interest

The authors declare no conflicts of interest.

## Supporting information




**Supporting File**: advs74172‐sup‐0001‐SuppMat.docx.

## Data Availability

The data that support the findings of this study are available from the corresponding author upon reasonable request.
