# Supplementary material for: Tim1 Deficiency Mediates Gestational Hyperglycemia‐Related Syncytiotrophoblast Dysfunction and Fetal Growth Restriction
Source: Adv Sci (Weinh). 2026 Feb 11;13(20):e08686. doi: 10.1002/advs.202508686 (PMC13067870; doi:10.1002/advs.202508686)
Supplement: Supplementary file 1 — Supporting File: advs74172‐sup‐0001‐SuppMat.docx. [file ADVS-13-e08686-s001.docx]

**Online Supplemental Material**

***Tim1* Deficiency Mediates Gestational Hyperglycemia-Related Syncytiotrophoblast Dysfunction and Fetal Growth Restriction**

**Title and page number of each element included in the document**

**Expanded Methods Page 2**

**Supplemental Tables Page12**

**Supplemental Figures Page14**

**Expanded Methods**

**Human Studies**

Placenta tissues were procured from deliveries at the Fourth Affiliated Hospital, Zhejiang University School of Medicine. These placenta tissues are intimately linked with clinical data in electronic medical records system. The study was approved by the Ethics Committee of the Fourth Affiliated Hospital, Zhejiang University School of Medicine (Approval No. K2024238), and informed consent has been garnered from all participants.

Placenta Sample Collection Procedure: Upon delivery of the placenta, a fragment of villous tissue measuring approximately 2 cm × 2 cm × 2 cm is excised from the midpoint between the umbilical cord attachment site and the placental margin. The villous tissue is subsequently washed in phosphate buffered saline (PBS) to eliminate as much blood as feasible. Subsequently, it is sectioned into smaller fragments and placed into cryovials. These cryovials are relocated to a -80°C ultra-low temperature freezer for prolonged storage.

We collected 40 placenta tissue samples. The case group encompassed pregnant women with pregestational diabetes (PGDM) and fetal growth restriction (FGR) (n = 20), whereas the control (CON) group consisted of normal pregnancies (n = 20).

Pregestational diabetes is diagnosed before pregnancy. However, it is often first recognized or discovered during the first trimester screening in women who were previously undiagnosed [1]. FGR was diagnosed according to the international Delphi consensus [2]. And the exclusion criteria included chronic diseases (hypertension, liver, kidney, heart, lung, and other major organ diseases), autoimmune diseases (Sjogren’s syndrome, anticardiolipin syndrome, myasthenia gravis), tumors, preeclampsia, preterm birth, intrahepatic cholestasis of pregnancy, any other identified obstetric disorders, and clearly defined genetic diseases in the fetus.

**Animal Care**

All animal procedures were performed in accordance with the protocol approved by the Animal Care and Use Committee of Zhejiang University. Adult female and male ICR mice (8 weeks old) were purchased from Shanghai SLAC Laboratory Animal Co. (Shanghai, China). Mice showing signs of disease, abnormal behaviors, fur loss, or body weight not meeting the criteria before the experiment were excluded. The mice were allowed 1 week to acclimate to the experimental environment in Laboratory Animal Center of Zhejiang University. All mice were housed under pathogen-free conditions at a temperature of 23°C under a 12-hour dark/light cycle. All mice were provided with the same standard chow and water. The day with the presence of a vaginal plug was defined as day 0.5 of embryonic development after fertilization (E0.5). After fasting for 12 hours, pregnant mice were divided into two groups by drawing lots, and received intraperitoneal injections of streptozotocin (STZ; 150 mg/kg) and sodium citrate buffer at E1.5, respectively. Pregnant mice were measured for blood glucose levels at E2.5, E4.5, E6.5, E8.5, E10.5, E12.5, E14.5, E16.5, and E18.5. Finally, outcome measures were assessed, including fetal crown-rump length, fetal weight, placental weight, placental efficiency, changes in the labyrinth zone, and alterations in syncytiotrophoblasts.

**Antioxidant Therapy in Mice**

The pregnant mice were divided into 4 groups: CON, GDM, GDM-NAC, and GDM-CoQ10. The CON group was defined the control group. In the CON group, the pregnant mice were not given any treatment. In the GDM group, the GDM model mice were not given any treatment. In the GDM-NAC group, the GDM model mice were orally given drinking water containing N-Acetylcysteine (NAC; 10 mg/ml; MCE, China, HY-B0215) from E2.5 to E18.5. In the GDM-CoQ10 group, the GDM model mice were treated with a 1% Coenzyme Q10 (CoQ10, MCE, China, HY-N0111) diet from E2.5 to E18.5.

**Generation of *Tim1* Knockout Mice**

*Tim1*^+/−^ C57BL/6J mice (Strain NO. T003304) were purchased from GemPharmatech (Nanjing, China). The heterozygous (HET) × HET crosses generated *Tim1*^−/−^ knockout (KO) mice. PCR genotyping was performed using tail DNA extraction. The pregnant mice were euthanized at specific times. The fetuses and placentas of pregnant mice were collected for the following experiments. The wild type (WT) was defined as the control group.

**Cell Culture**

BeWo cells (CL-0500) were kindly provided by Procell Life Science&Technology Co.,Ltd. BeWo cells were cultured in Ham’s F12K medium (HyClone, SH30526.01) supplemented with 10% fetal bovine serum (Gibco, A5669701) and 1% penicillin / streptomycin (Gibco, 10378016). All cells were cultured in a humidified atmosphere with 5% CO2 at 37°C.

**Isolation and culture of** **primary human trophoblasts**

The minced placenta tissues were digested with trypsin (Sigma Aldrich, MO, USA) and DNase I (Sigma Aldrich). The supernatant was centrifuged, and the cell suspension was separated by Percoll (GE Healthcare BioSciences AB, Uppsala, Sweden) density gradient. Primary human trophoblasts cells were collected from the density layer at 30-50% and cultured in Ham’s F12: DMEM media (HyClone, CT, USA) with 10% fetal bovine serum (FBS; GIBCO, MA, USA) and antibiotics in a 5% CO2 air incubator at 37°C. Cells were then maintained for 72 h after plating.

**Cell Fusion Assay**

Previous studies have described methods for quantifying cell fusion [3,4]. Syncytialization of BeWo cells was induced with forskolin (FSK). Briefly, BeWo cells were seeded on poly-L-lysine–coated coverslips for 24 hours at 5% CO_2_ and 37°C. After 24 hours, BeWo cells were treated with 40 μM FSK (MCE, HY-15371) for 48 hours to induce cell fusion. The culture medium containing 40 μM FSK was replaced every 24 hours. Cells were fixed with methanol pre-cooled to -20℃, then incubated at 4℃ overnight with anti-E-cadherin antibody (1:150, Abcam, ab40772). This was followed by incubation with Alexa Fluor 594–goat anti-rabbit IgG (H+L) antibody (1:150, BIOKER, BK-R594) at room temperature for 1.5 hours. Samples were counterstained with 5 μg/mL 4',6-diamidino-2-phenylindole (DAPI) at room temperature for 15 minutes, mounted with antifading mounting medium (Solarbio, S2100). For each sample, fluorescent images were randomly captured at least six fields using OLYMPUS FV3000, OSR inverted confocal microscope using a ×20 objective. ImageJ was used for imaging analysis. Cells with more than two nuclei were defined as fused cells. To quantify cell fusion, fusion index of each sample was calculated as the ratio of the number of nuclei from fused cells over the total number of nuclei.

**Determination of Cellular Oxidative Stress**

Dihydroethidium (DHE; MCE, HY-D0079) staining was used to detect the level of cellular reactive oxygen species (ROS). BeWo cells were cultured in medium containing D-Glucose (D-Glu; 5mM; Thermo Fisher, A2494001), D-Glu (35mM), D-Glu (35mM) + NAC (5mM) and D-Glu (35mM) + CoQ10 (10μM). Subsequently, the cells were incubated with 1μM DHE solution at 37°C for 30 minutes. Finally, fluorescent images were randomly captured using an OLYMPUS FV3000, OSR inverted confocal microscope with a 20x objective.

**Placenta Histology Analysis**

Placentas were fixed in 4% paraformaldehyde for 24 hours, dehydrated in ethanol, and embedded in paraffin. Following standardized protocols [5,6], we consistently sectioned the placenta at the sagittal midline, using the site of umbilical cord insertion as a landmark to ensure the section was representative of the overall placental structure. At this location, each placenta was serially sectioned at 5 µm thickness and stained with hematoxylin and eosin (H&E). Sections were visualized using an Olympus VS200 scanner, and regional distributions within the placenta were analyzed. This method ensures consistency and reproducibility in sampling, enabling accurate comparisons between treatment groups. For immunohistochemistry, the placental tissue was sectioned at a thickness of 5 µm, and the sections were incubated separately with anti-TIM1 (1:200, Abcam, ab78494) antibodies. Stained sections were visualized using OLYMPUS VS200. Immunofluorescence staining against *Mct1*, *Mct4* and *Tim1* was performed on placentas using anti-MCT1 antibody (1:100, Millipore-Sigma, AB1286-I), anti-MCT4 antibody (1:100, Santa Cruz Biotechnology Inc., sc-376140) and anti-TIM1 antibody (1:200, Abcam, ab78494). Fluorescent images were randomly captured with OLYMPUS FV3000, OSR inverted confocal microscope using a 20× objective. Antibodies are listed in **Supplemental Table 2**.

**Knockdown Using Specific siRNA**

BeWo cells were transiently transfected using Lipofectamine™ RNAiMAX (Thermo Scientific, 13778150) with specific siRNA targeting human *TIM1* or nonspecific scramble siRNA as a negative control. Following transfection, the medium was replaced with fresh F-12K medium supplemented with 10% FBS. The cells were harvested for further analysis 48 hours post-transfection. The siRNAs targeting *TIM1* were designed and synthesized by RiboBio (Guangzhou, China). The sequences of these siRNAs are listed in **Supplemental Table 3.**

**Overexpression of TIM1**

TIM1 overexpression lentiviruses were constructed by GenePharma Co., Ltd (Shanghai, China). The overexpression lentivirus and its vectors were added to the culture medium. Eight to twelve hours later, the lentivirus was removed, and fresh culture medium was added.

**Quantitative Real-time PCR**

Total RNA was isolated from the whole placenta tissues or cells using RNAiso Plus (TaKaRa, 9108). Reverse transcription was performed using the PrimeScript™ RT Master Mix (TaKaRa, RR036A). TB Green® Premix Ex Taq™ (TaKaRa, RR420A) was then used. The relative quantification for each mRNA was calculated using the 2−ΔΔCt method, with *β-Actin* as an internal reference. For each independent biological samples, the qPCR reaction was performed in three technical triplicates, and the mean value of these technical replicates was used for the statistical analysis. The primers and gene names are listed in **Supplemental Table 4,** and their specificity was verified using BLAST.

**Western Blot**

The whole placenta tissues or cells were homogenized and placed in RIPA Lysis Buffer (Beyotime, P0013B) to obtain protein samples. The total protein concentration in the samples was measured using the BCA Protein Assay Kit (Thermo Scientific, 23225). Protein samples were loaded and electrophoresed on polyacrylamide gels, and the separated proteins were then transferred onto the PVDF membrane. The membranes were blocked with 5% non-fat milk in Tris-Borate-Sodium Tween-20 (TBST) buffer for 2 hours and incubated with primary antibodies against TIM1 (1:2000, Zenbio, R382387), β-ACTIN (1:3000, BIOKER, BK7018) and GAPDH (1:3000, BIOKER, BK7021) at 4°C overnight. Afterward, the membranes were washed and incubated with an HRP-conjugated secondary antibody for 1 hour at room temperature. The bands were visualized by chemiluminescence using an ECL kit (Share-Bio, SB-WB012). The density of the target protein, calculated using ImageJ software, was normalized to GAPDH. All antibodies used are listed in **Supplemental Table 2**.

**Transmission Electron Microscope**

Samples were fixed in 2.5% glutaraldehyde in 0.1 M PBS buffer (pH 7.4) at 4°C overnight. Next, samples were fixed in 1% osmium tetroxide for 1 hour and embedded in epoxy resin. Samples were sectioned to a thickness of 60 nm and stained with uranyl acetate and lead citrate. Images were acquired using a Talos L120C transmission electron microscope (Thermo Fisher Scientific).

**RNA Sequencing**

RNA high-throughput sequencing was performed by Cloud-Seq Biotech (Shanghai, China). Potential confounding variables were minimized by maintaining consistent housing conditions, diet and water supply, strain, random group allocation, and inclusion of a vehicle control for injections. Total RNA was extracted from the whole placenta. Briefly, total RNA was used to remove rRNAs with the GenSeq® rRNA Removal Kit (GenSeq, Inc.). The rRNA-depleted samples were then subjected to library construction with the GenSeq® Low Input RNA Library Prep Kit (GenSeq, Inc.) according to the manufacturer’s instructions. Libraries were quality-controlled and quantified using the BioAnalyzer 2100 system (Agilent Technologies, Inc., USA). Library sequencing was performed on the Illumina NovaSeq instrument with 150 bp paired-end reads. Paired-end reads were harvested from the Illumina NovaSeq 6000 sequencer and quality-controlled at Q30. After 3’ adaptor-trimming and removal of low-quality reads with Cutadapt software (v1.9.3), the high-quality clean reads were aligned to the reference genome using HISAT2 software (v2.0.4). HTSeq software (v0.9.1) was used to obtain raw counts. Differentially expressed mRNAs were then identified based on p-value and fold change (fold change ≥2 with adjusted p-value ≤0.01).

**Reference**

[1] American College of Obstetricians and Gynecologists’ Committee on Practice Bulletins—Obstetrics, ACOG Practice Bulletin No. 201: Pregestational Diabetes Mellitus, Obstet. Gynecol. 132 (2018) e228–e248. https://doi.org/10.1097/AOG.0000000000002960.

[2] C.C. Lees, T. Stampalija, A.A. Baschat, F. Da Silva Costa, E. Ferrazzi, F. Figueras, K. Hecher, J. Kingdom, L.C. Poon, L.J. Salomon, J. Unterscheider, ISUOG Practice Guidelines: diagnosis and management of small‐for‐gestational‐age fetus and fetal growth restriction, Ultrasound Obstet. Gynecol. 56 (2020) 298–312. https://doi.org/10.1002/uog.22134.

[3] Y. Zhang, T. Le, R. Grabau, Z. Mohseni, H. Kim, D.R. Natale, L. Feng, H. Pan, H. Yang, TMEM16F phospholipid scramblase mediates trophoblast fusion and placental development, Sci. Adv. 6 (2020) eaba0310. https://doi.org/10.1126/sciadv.aba0310.

[4] X. Shao, G. Cao, D. Chen, J. Liu, B. Yu, M. Liu, Y.-X. Li, B. Cao, Y. Sadovsky, Y.-L. Wang, Placental trophoblast syncytialization potentiates macropinocytosis via mTOR signaling to adapt to reduced amino acid supply, Proc. Natl. Acad. Sci. 118 (2021) e2017092118. https://doi.org/10.1073/pnas.2017092118.

[5] V. Perez-Garcia, E. Fineberg, R. Wilson, A. Murray, C.I. Mazzeo, C. Tudor, A. Sienerth, J.K. White, E. Tuck, E.J. Ryder, D. Gleeson, E. Siragher, H. Wardle-Jones, N. Staudt, N. Wali, J. Collins, S. Geyer, E.M. Busch-Nentwich, A. Galli, J.C. Smith, E. Robertson, D.J. Adams, W.J. Weninger, T. Mohun, M. Hemberger, Placentation defects are highly prevalent in embryonic lethal mouse mutants, Nature 555 (2018) 463–468. https://doi.org/10.1038/nature26002.

[6] S. Andrews, C. Krueger, M. Mellado-Lopez, M. Hemberger, W. Dean, V. Perez-Garcia, C.W. Hanna, Mechanisms and function of de novo DNA methylation in placental development reveals an essential role for DNMT3B, Nat. Commun. 14 (2023) 371. https://doi.org/10.1038/s41467-023-36019-9.

**Supplemental Table 1. General characteristics of the human participants**

|  |  | Normal Pregnancy (n = 20) | PGDM - FGR (n = 20) | P |
| --- | --- | --- | --- | --- |
| Age (Years) | | 29.55 ± 5.63 | 30.40 ± 5.09 | 0.62 |
| Pre-pregnancy BMI (kg/m2) | | 21.26 ± 1.82 | 26.12 ± 1.99 | <0.001 |
| Gravidity | | 2.85 ± 0.88 | 2.50 ± 1.19 | 0.43 |
| Parity | | 1.45 ± 0.60 | 1.30 ± 0.47 | 0.46 |
| HbA1c (%) | | 4.80 ± 0.60 | 6.63 ± 0.31 | <0.001 |
| Fasting blood glucose  in 1st trimester | | 4.65 ± 0.70 | 7.70 ± 0.59 | <0.001 |
| Gestational age (week) | | 38.05 ± 1.00 | 37.65 ± 0.81 | 0.2 |
| Birth weight (g) | | 3279.50 ± 374.82 | 2290.75 ± 166.69 | <0.001 |
| Sex | |  |  | 0.75 |
|  | Male | 11 (55%) | 13 (65%) |  |
|  | Female | 9 (45%) | 7 (35%) |  |

Data are shown as mean ± SD. Age, Pre-pregnancy BMI, and fasting blood glucose in first trimester were compared by unpaired t test. Gravidity, parity, HbA1c, gestational age and birth weight were compared by Mann-Whitney U test. Sex was compared by the chi-square test. BMI, body mass index; HbA1c, hemoglobin A1c.

**Supplemental Table 2. Antibodies used in this study**

| Antibody | Host species | Catalogue number | Manufacturer | Final concentration |
| --- | --- | --- | --- | --- |
| Mct1 | Chicken | AB1286-I | Millipore-Sigma | 1:200 (IF) |
| Mct4 | Mouse | sc-376140 | Santa Cruz | 1:100 (IF) |
| TIM1 | Rabbit | ab78494 | Abcam | 1:150 (IF/IHC) |
| E-Cadherin | Rabbit | ab40772 | Abcam | 1:150 (IF) |
| GAPDH | Rabbit | BK7021 | BIOKER | 1:3000 (WB) |
| β-ACTIN | Rabbit | BK7018 | BIOKER | 1:3000 (WB) |
| TIM1 | Rabbit | R382387 | Zenbio | 1:2000 (WB) |

**Supplemental Table 3. The sequences of siRNAs**

| siRNAs | Sequences (5'-3') |
| --- | --- |
| siTIM1_001 | GACGGCCAATACCACTAAA |
| siTIM1_002 | GGCTCATGTTCTCTATTCA |
| siTIM1_003 | CGACTGTTCTGACGACAAT |

**Supplemental Table 4. Sequence of the primers used for real-time qPCR.**

| Gene | Forward Primer | Reverse Primer |
| --- | --- | --- |
| Mus musculus |  |  |
| Actb | GGCTGTATTCCCCTCCATCG | CCAGTTGGTAACAATGCCATGT |
| Mct1 | AGTGCAACGACCAGTGAAGT | GCGATCATTACTGGACGGCT |
| Mct4 | ATACAGCGGCTGGCGGTAA | GCTGCTTTCACCAAGAACTGA |
| Stat1 | TCACAGTGGTTCGAGCTTCAG | GCAAACGAGACATCATAGGCA |
| Tim1 | ACATATCGTGGAATCACAACGAC | ACAAGCAGAAGATGGGCATTG |
| Homo Sapiens |  |  |
| GAPDH | ACAACTTTGGTATCGTGGAAGG | GCCATCACGCCACAGTTTC |
| TIM1 | TGGCAGATTCTGTAGCTGGTT | AGAGAACATGAGCCTCTATTCCA |

**Supplemental Figures and Legends**


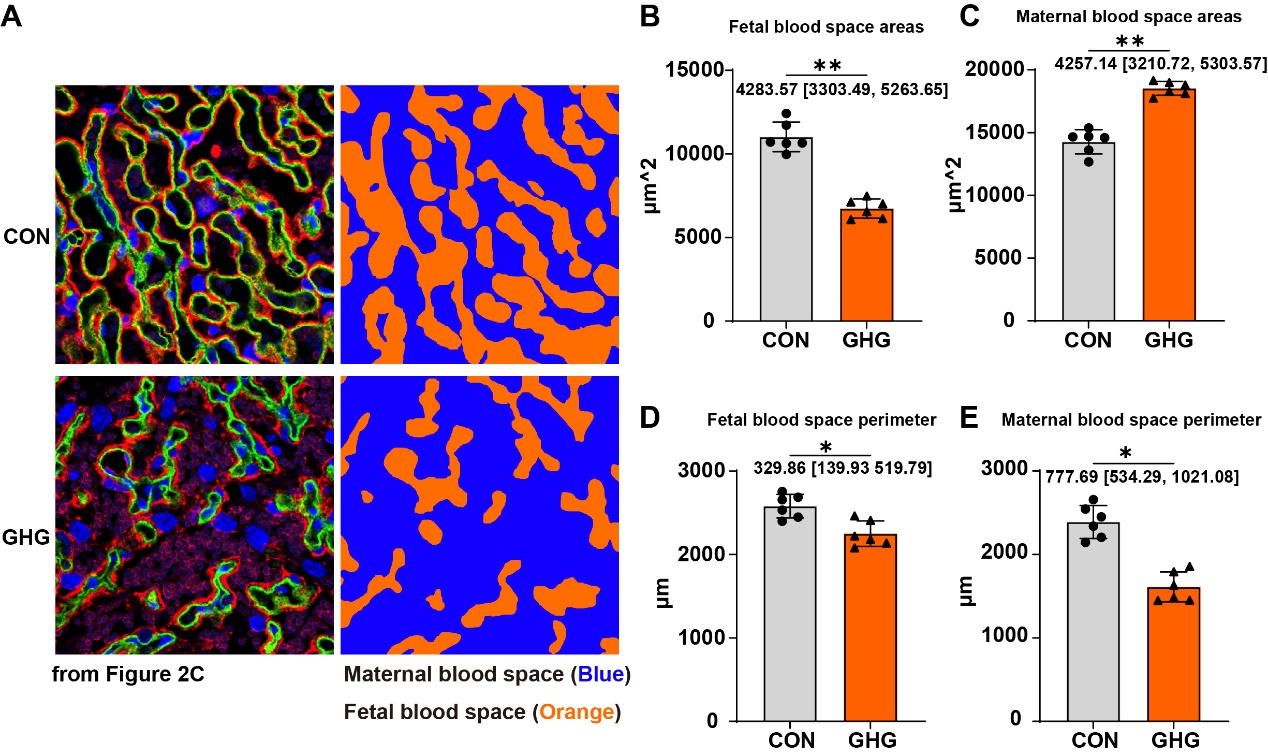


**Supplemental Figure 1. Analysis of placental vascularization in the placenta of mice with gestational hyperglycemia.** (**A**) Representative image of fetal and maternal blood spaces as determined by MCT4 (Green)- and MCT1 (Red)- enclosed spaces, respectively. (**B-E**) Quantification of fetal blood space area (**B**), maternal blood space area (**C**), fetal blood space perimeter (**D**), maternal blood space perimeter (**E**) (n = 6 individual experiments per group). Data are presented as mean ± SD. Multiple placentas from the same dam were considered non-independent and analyzed using a linear mixed model with Dam as a random intercept (**B–E**). The mean difference between the two groups, along with the 95% confidence interval, is displayed beneath the significance bar. “*n*” indicates the number of independent biological replicates; technical replicates are averaged within each biological replicate before statistical analysis and are not included in the “*n*” values. P < 0.05 was considered statistically significant. *P < 0.01, **P < 0.001.


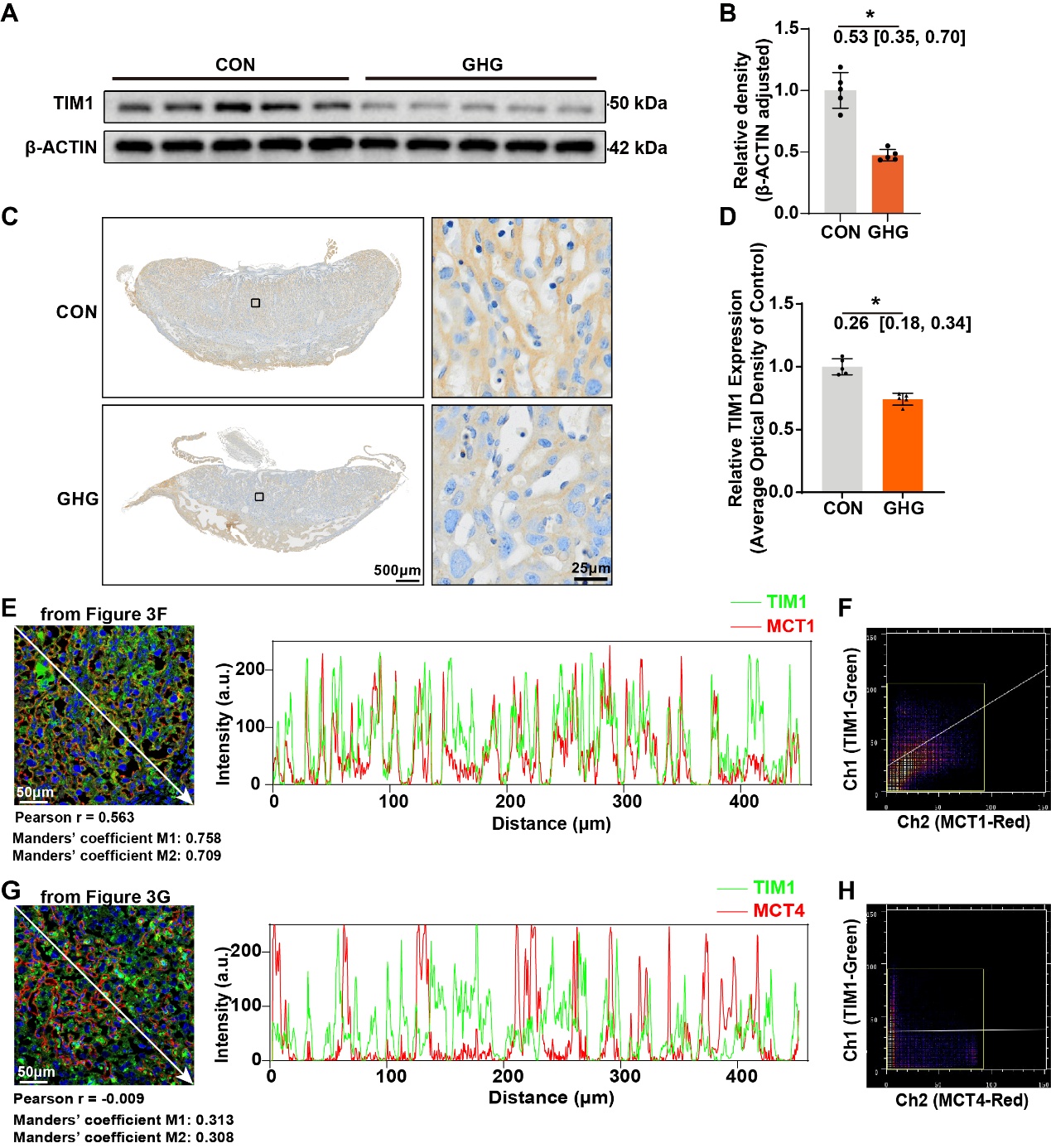


**Supplemental Figure 2.** (**A**) Representative Western blot image showing TIM1 protein expression in mouse placentas. (**B**) Quantification of TIM1 protein expression in mouse placentas (n = 5 individual experiments per group). (**C**) Representative images of immunohistochemistry (IHC) staining for TIM1 expression in mouse placentas. (**D**) Quantification of TIM1 expression in mouse placentas by IHC (n = 5 individual experiments per group). (**E**) Intensity profiles show colocalization of TIM1 (green) and MCT1 (red) along the direction of the white arrow. (**F**) Colocalization scatter plot of TIM1 and MCT1 (Pearson coefficient: 0.563, Manders’ coefficient M1: 0.758, Manders’ coefficient M2: 0.709). (**G**) Intensity profiles show colocalization of TIM1 (green) and MCT4 (red) along the direction of the white arrow. (**H**) Colocalization scatter plot of TIM1 and MCT4 (Pearson coefficient -0.009, Manders’ coefficient M1: 0.313, Manders’ coefficient M2: 0.308). Data are presented as mean ± SD. Multiple placentas from the same dam were considered non-independent and analyzed using a linear mixed model with Dam as a random intercept (**B** and **D**). The mean difference between the two groups, along with the 95% confidence interval, is displayed beneath the significance bar. “*n*” indicates the number of independent biological replicates; technical replicates are averaged within each biological replicate before statistical analysis and are not included in the “*n*” values. P < 0.05 was considered statistically significant. *P < 0.001.


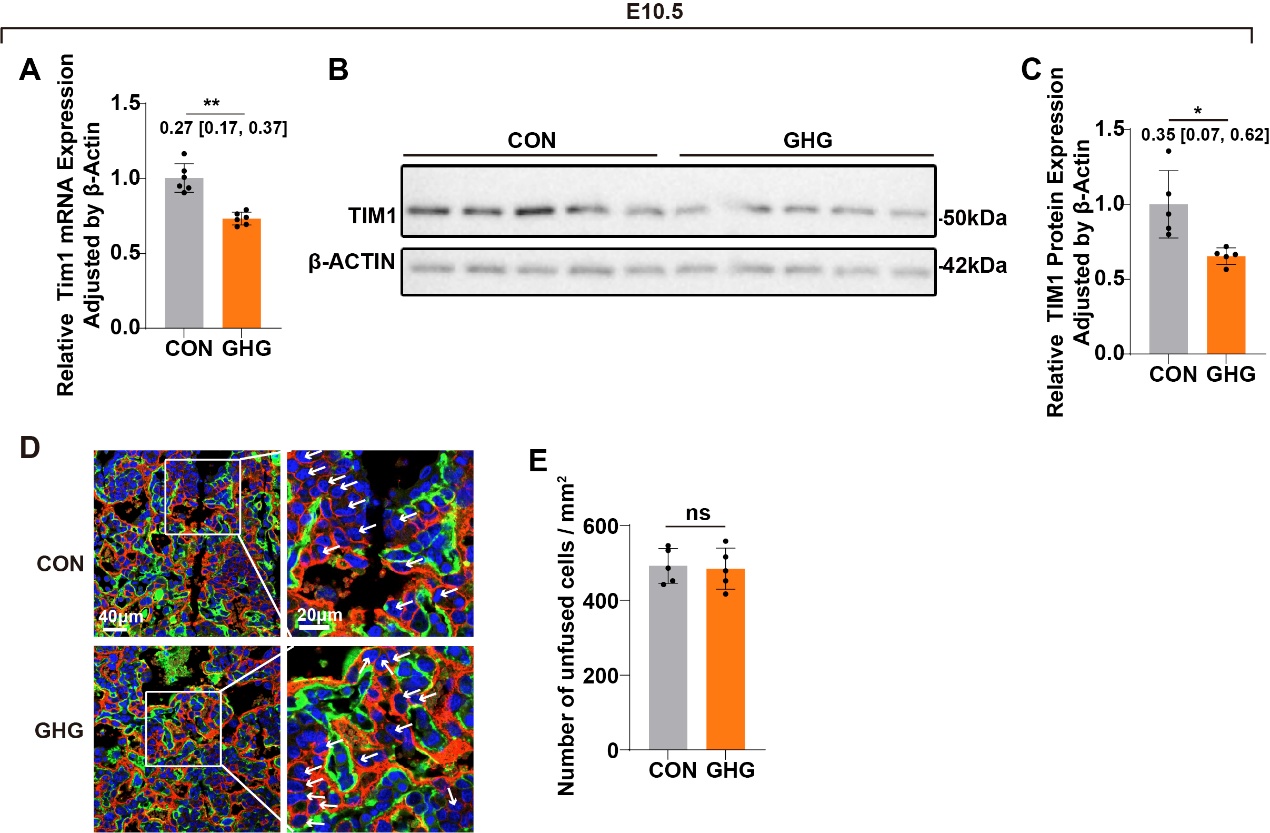


**Supplemental Figure 3. (A)** The mRNA expression levels of *Tim1* in E10.5 placentas from the CON and GHG groups (n = 6 placentas per group). (**B**) Representative Western blot image showing TIM1 protein expression in E10.5 placentas. (**C**) Quantification of TIM1 protein expression in E10.5 placentas (n = 5 placentas per group). (**D**) Immunofluorescence for MCT1 and MCT4 in E10.5 placentas from the CON and GHG groups. Arrows indicate unfused trophoblast cells. (**E**) Quantification of unfused trophoblast cells in E10.5 placentas from the CON and GHG groups (n = 5 individual experiments per group). Data are presented as mean ± SD. Multiple placentas from the same dam were considered non-independent and analyzed using a linear mixed model with Dam as a random intercept (**A**, **C**, and **E**). The mean difference between the two groups, along with the 95% confidence interval, is displayed beneath the significance bar. “*n*” indicates the number of independent biological replicates; technical replicates are averaged within each biological replicate before statistical analysis and are not included in the “*n*” values. P < 0.05 was considered statistically significant. *P < 0.05, **P < 0.001.


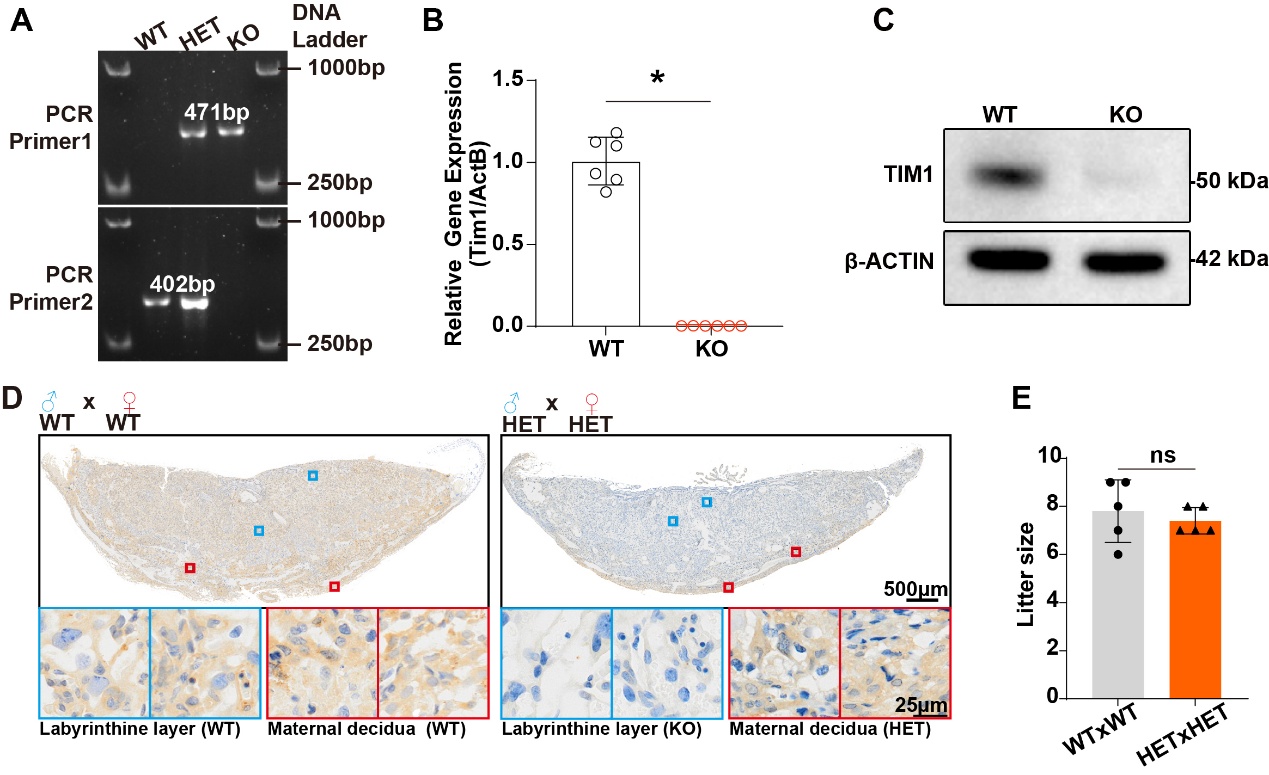


**Supplemental Figure 4.** (**A**) Genotyping PCR of DNA from wild-type (WT, *Tim1*^+/+^), heterozygous (Het, *Tim1*^+/-^), and homozygous (KO, *Tim1*^-/-^) fetal mice. (**B**) qPCR showing the Tim1 mRNA expression in fetal mouse tissues with WT and KO genotypes (n = 6 per group). (**C**) Western blot showing the TIM1 protein expression in fetal mouse tissues with WT and KO genotypes. (**D**) Representative TIM1 immunohistochemistry images of a WT placenta from a WT dam and a KO placenta from a HET dam, showing TIM1 expression in the labyrinth layer and decidua, respectively. (**E**) Bar plots showing litter size between the WT x WT and HET x HET breeding scheme (n = 5 per group). Data are presented as mean ± SD. Data were analyzed using Mann-Whitney U (**B**), and unpaired Student's t-test (**E**). The mean difference between the two groups, along with the 95% confidence interval, is displayed beneath the significance bar. “*n*” indicates the number of independent biological replicates; technical replicates are averaged within each biological replicate before statistical analysis and are not included in the “*n*” values. P < 0.05 was considered statistically significant. *P < 0.001.


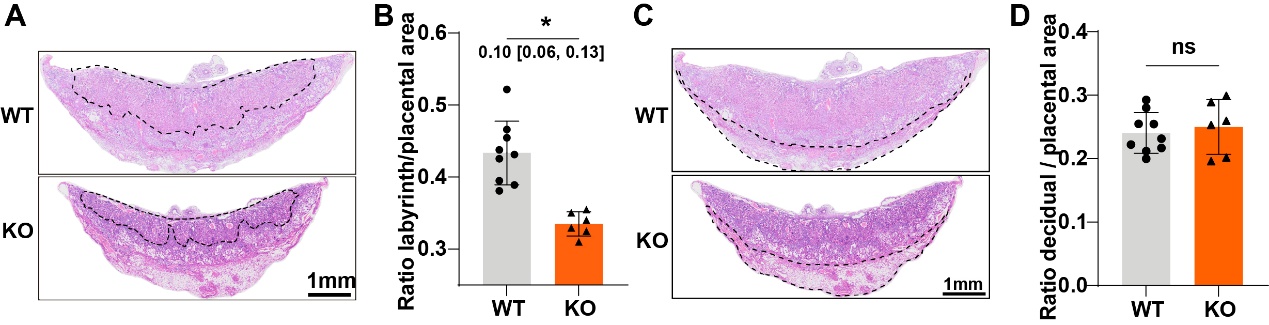


**Supplemental Figure 5. Tim1 KO induces impaired syncytiotrophoblast formation.** (**A**) Representative H&E staining images of E14.5 placentas. The dashed outlined region corresponds to the placental labyrinth zone. (**B**) Bar plot showing the ratio of labyrinth area to total placental area (n = 6-9 individual experiments per group). (**C**) Representative H&E staining images of E14.5 placentas. The dashed outlined region corresponds to the decidua. (**D**) Bar plot showing the ratio of decidua area to total placental area (n = 6-9 individual experiments per group). Data are presented as mean ± SD. Multiple placentas from the same dam were considered non-independent and analyzed using a linear mixed model with Dam as a random intercept (**B** and **D**). The mean difference between the two groups, along with the 95% confidence interval, is displayed beneath the significance bar. “*n*” indicates the number of independent biological replicates; technical replicates are averaged within each biological replicate before statistical analysis and are not included in the “*n*” values. P < 0.05 was considered statistically significant. *P < 0.05.


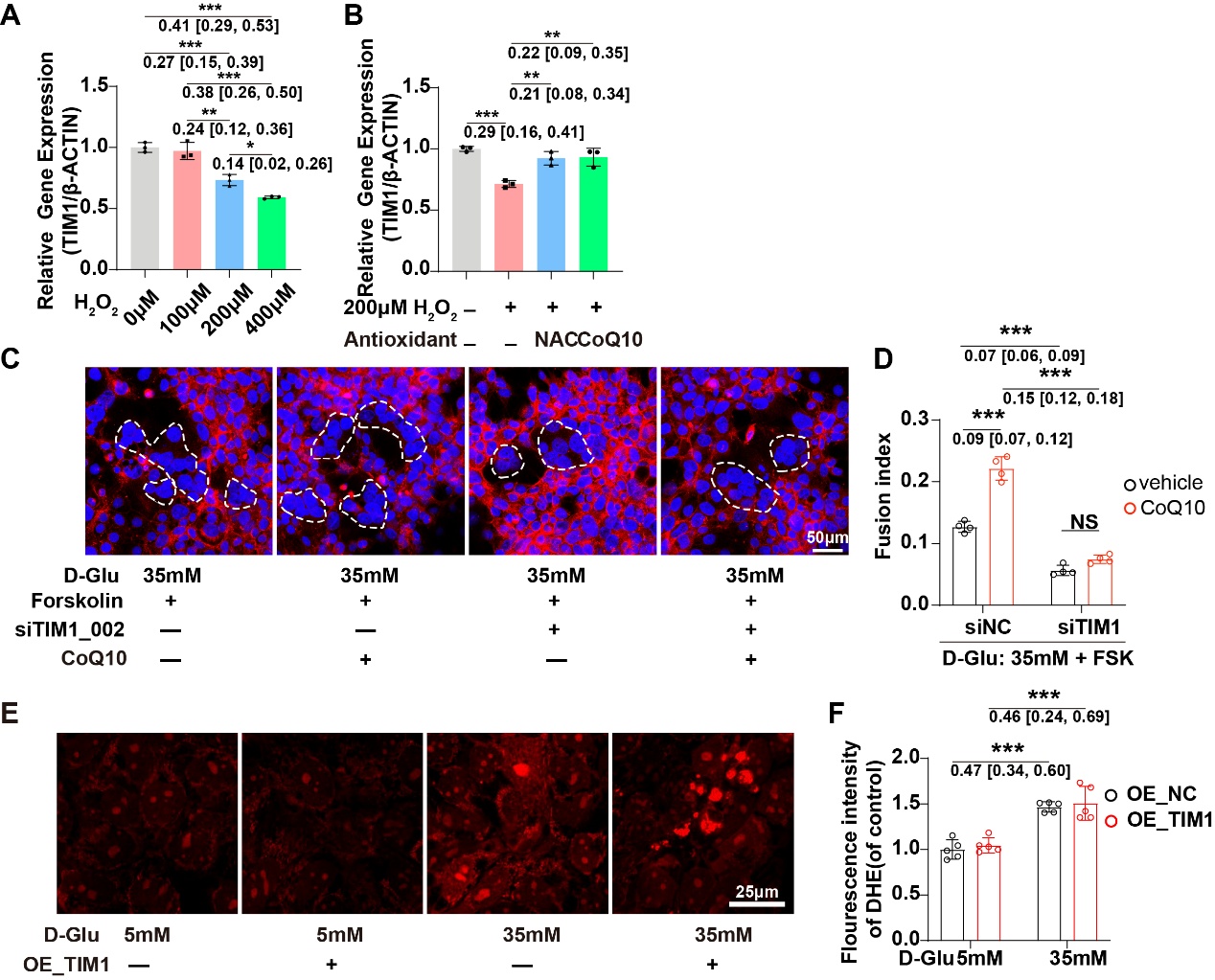


**Supplemental Figure 6.** (**A**) *Tim1* mRNA expression in BeWo cells treated with H_2_O_2_ (0 μM, 100 μM, 200 μM, 400 μM) (n = 3 individual experiments per group). (**B**) *Tim1* mRNA expression in BeWo cells treated with NAC (5 mM) or CoQ10 (10 μM) under H_2_O_2_ (200 μM) conditions (n = 3 individual experiments per group). (**C**) Cells were cultured under high glucose conditions and transfected with either control siRNA or TIM1 siRNA. Meanwhile, cells were treated with CoQ10 or vehicle, followed by assessment of cell fusion. White dotted lines indicate multinucleated cells. (**D**) Quantification of the fusion index in BeWo cells (n = 4 individual experiments per group). (**E**) Dihydroethidium (DHE) staining to measure steady-state ROS levels. (**F**) Quantification of DHE fluorescence intensity (n = 5 individual experiments per group). Data are presented as mean ± SD. Data were analyzed using one-way ANOVA followed by Tukey post hoc analysis (**A** and **B**), and two-way ANOVA followed by Tukey post hoc analysis (**D and F**). The mean difference between the two groups, along with the 95% confidence interval, is displayed beneath the significance bar. “*n*” indicates the number of independent biological replicates; technical replicates are averaged within each biological replicate before statistical analysis and are not included in the “*n*” values. P < 0.05 was considered statistically significant. *P < 0.05, **P < 0.01, ***P < 0.001.


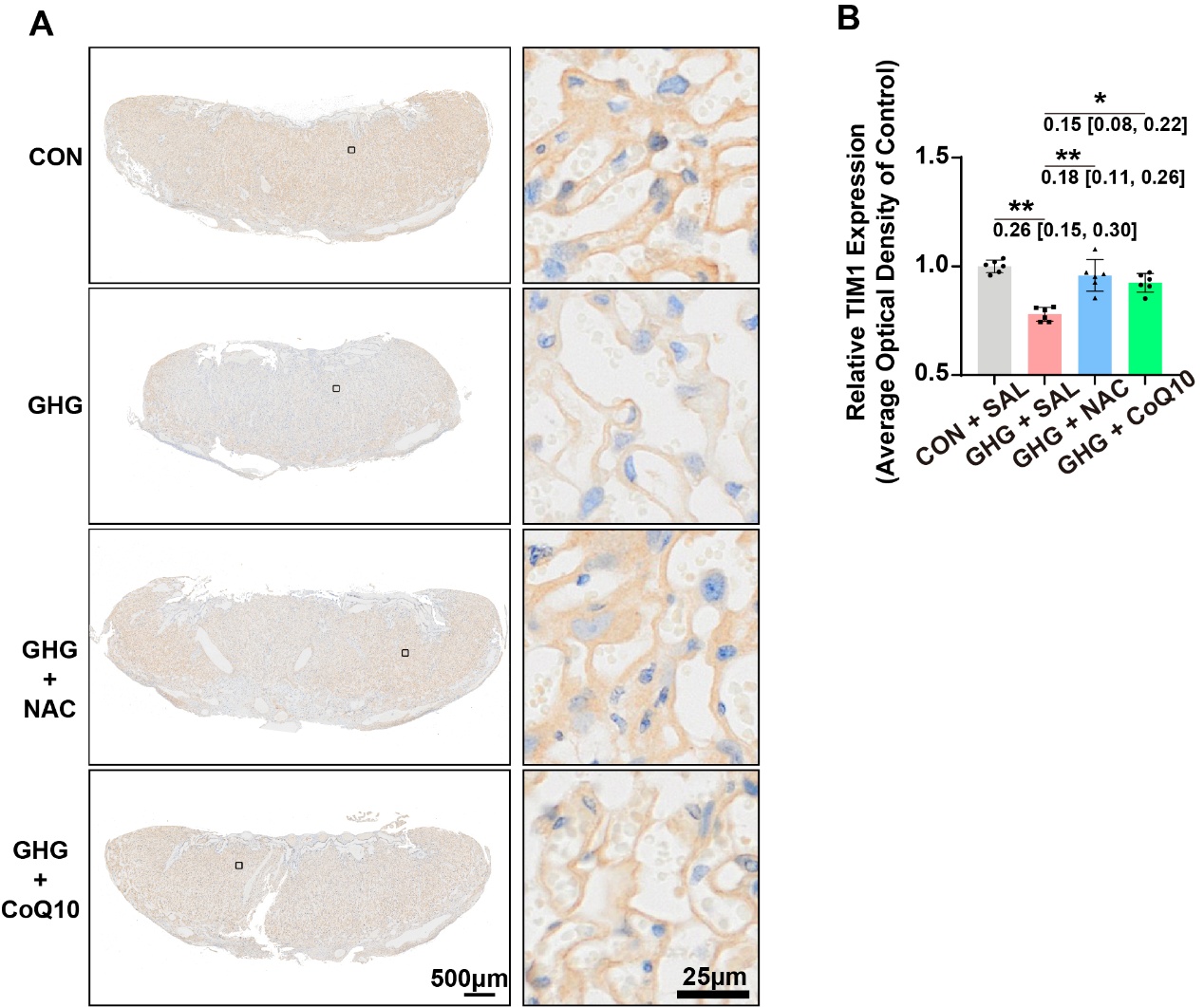


**Supplemental Figure 7.** (**A**) Representative images of immunohistochemistry staining for TIM1 expression in mouse placentas. (**B**) Quantification of TIM1 expression in mouse placentas by IHC (n = 8 individual experiments per group). Data are presented as mean ± SD. Multiple placentas from the same dam were considered non-independent and analyzed using a linear mixed model with Dam as a random intercept (**B**). The mean difference between the two groups, along with the 95% confidence interval, is displayed beneath the significance bar. “*n*” indicates the number of independent biological replicates; technical replicates are averaged within each biological replicate before statistical analysis and are not included in the “*n*” values. P < 0.05 was considered statistically significant. *P < 0.01, **P < 0.001.


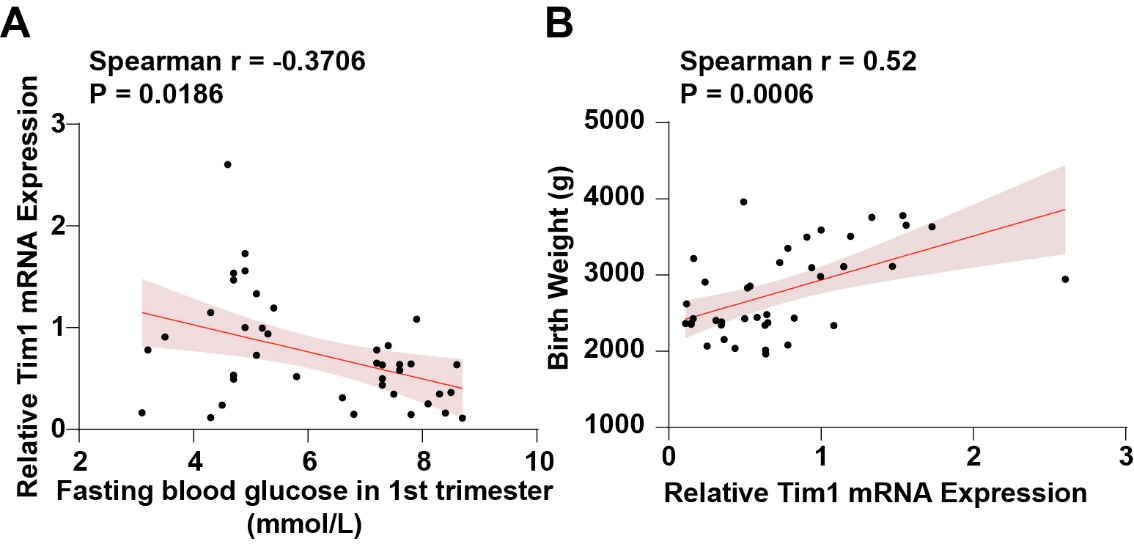


**Supplemental Figure 8 Correlations between placental *Tim1* mRNA expression and clinical data including first-trimester fasting blood glucose and birth weight.** (**A**) Scatter plot of 40 individual data points indicating a moderate negative correlation between maternal fasting blood glucose in the first trimester and relative placental *Tim1* mRNA expression (Spearman R = −0.3706, p = 0.0186). (**B**) Scatter plot of 40 individual data points showing a moderate positive correlation between relative placental *Tim1* mRNA expression and neonatal birth weight (Spearman R = 0.5200, p = 0.0006). P < 0.05 was considered statistically significant.
